# Fine mapping of early adipogenesis identifies committed progenitors with dual white and beige potential

**DOI:** 10.1016/j.isci.2026.118086

**Published:** 2026-10-29

**Authors:** Sarah Hazell Pickering, Damien Dufour, Natalia M. Galigniana, Philippe Collas, Nolwenn Briand

**Affiliations:** 1 Department of Molecular Medicine, Institute of Basic Medical Sciences, Faculty of Medicine, University of Oslo, 0317 Oslo, Norway; 2 Department of Immunology and Transfusion Medicine, Oslo University Hospital, 0372 Oslo, Norway

**Keywords:** human adipose stem and progenitor cells, adipogenesis, beige adipocyte, single-cell RNA-seq, thermogenesis, beiging

## Abstract

Thermogenic capacity in adipose tissue varies among individuals and declines with age, obesity, and insulin resistance. To investigate the cellular basis of this variability, we performed parallel white and beige adipogenic differentiation using subcutaneous adipose progenitors from six healthy subjects. Beige adipogenesis elicits subject-specific, UCP1-dependent, and non-canonical thermogenic programs. Single-cell transcriptomics reveals varied proportions of progenitor populations, defined by differential expression of extracellular matrix, cell adhesion, and cytoskeletal genes. Mapping adipogenic commitment uncovers a continuum of transcriptional states largely shared between white and beige differentiation. Within this continuum, we identify ICAM1-positive committed progenitors contributing to both white and beige phenotypes. Beige induction increases the proportion of structural adipose tissue-resident cells with distinct extracellular matrix signatures, suggesting that early microenvironmental remodeling primes plasticity in mature cells. These results support a model in which diverse adipocyte fates arise from plastic progenitors, with individual differences in thermogenic adaptation driven by genetic, environmental, and epigenetic factors.

## Highlights

• Beige induction elicits subject-specific thermogenic programs
• Single cell maps reveal shared early states during white and beige adipogenesis
• ICAM1^high^ progenitors display dual white and beige differentiation potential

## Graphical abstract

**Figure:**
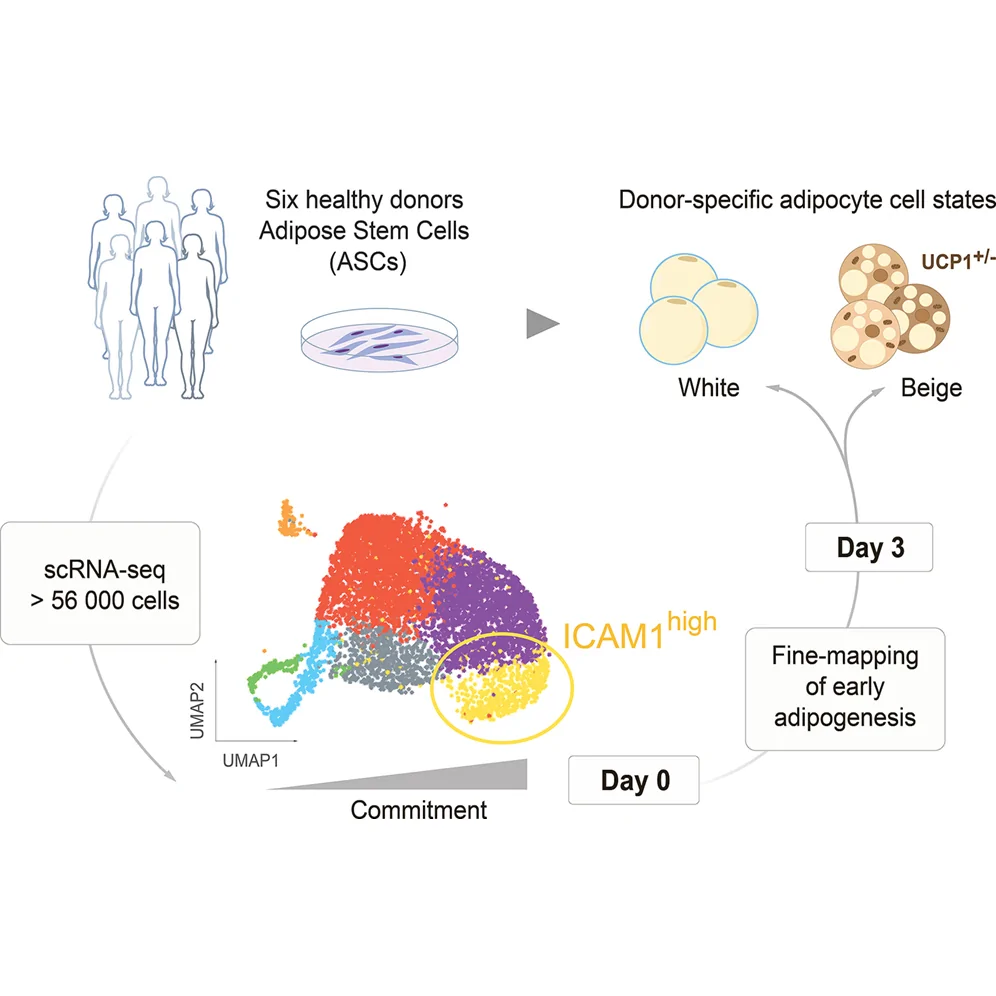

## Teaser

Cell biology; Developmental biology; Transcriptomics

## Introduction

Adipose tissue is a key player in whole-body energy homeostasis, responding dynamically to caloric flux and environmental temperature. Plasticity within white adipose tissue can influence metabolic outcomes, either mitigating or contributing to the development of metabolic disorders such as obesity and type 2 diabetes.^1^ Subcutaneous white adipose tissue contributes positively to metabolic health by playing a key role in endocrine signaling and the absorption of excess calories, thus preventing lipotoxicity in non-adipose organs.^2^ Adipose tissue thermogenesis, which can be induced by environmental cold, exercise, or dietary interventions, also correlates with a healthy, lean phenotype in adults.^3^ Activated beige adipocytes perform thermogenesis in white adipose tissue, utilizing energy-rich substrates which contribute to body-wide metabolic flux.^4^ Heterogeneous mechanisms, such as canonical UCP1 uncoupling, creatine and lipid futile cycling contribute independently or in combination to thermogenesis at the molecular level.^5^^,^^6^ Although adipose tissue thermogenesis is inducible in lean and healthy obese adults,^7^^,^^8^ thermogenic activity decreases with age and insulin resistance.^9^ Therefore, what underlies variation in human thermogenic phenotypes, and why individuals with adipose dysfunction tend to lack active beige adipocytes, remain key questions in the field.

Beige adipocytes have been reported to both differentiate *de novo* from progenitor cells and trans-differentiate from white adipocytes. However, it is unclear whether *de novo* differentiation occurs from a beige-specific subpopulation, as suggested by some murine studies.^10^^,^^11^^,^^12^ In mice, adipose progenitors are broadly classified as proliferative (DPP4+) or committed (ICAM+).^13^ In addition, it has been suggested that beige adipocytes originate from progenitors expressing smooth muscle markers such as *Acta2 and Sm22*,^11^ or from UCP1+ progenitor cells.^10^ Yet, there is minimal overlap in progenitor markers between mouse and human studies.^14^ Therefore, elucidating the mechanisms contributing to the differentiation of human white vs. beige adipocytes remains a challenge. Whether adipose progenitors are lineage-restricted toward white or beige fates, or whether beige identity reflects a plastic, inducible state acquired during differentiation, is an ongoing debate.^15^

High-resolution insights into beige and white adipogenesis in humans have been enabled by single-cell RNA sequencing (scRNA-seq) studies on extracted tissue and cultured adipose stem cells (hASCs). Meta studies of adipose tissue progenitor populations have identified a range of cell states,^16^^,^^17^ although it is unclear whether these reflect the original localization of cells within the tissue, or intrinsic lineage-specific priming. Few progenitor cell states have been reported in cultured hASCs,^18^ with adipogenic stimulation eliciting two trajectories: (1) structural WNT-regulated adipose tissue (SWAT) cells which remain multipotent via active WNT2 signaling and strongly express extracellular matrix (ECM) components (e.g., *DCN* and *MGP*) and (2) an adipogenic cell population which expresses lipid metabolism genes (e.g., *ADIPOQ* and *FABP4*), similar to preadipocytes from *in vivo* studies.^18^^,^^19^ *De novo* adipogenesis from deep neck progenitors elicits earlier activation of lipid metabolism compared to subcutaneous progenitors^20^; however, the mechanisms governing beige adipogenesis within the subcutaneous adipose depot have yet to be explored.

Despite the rapid growth of single-cell and single-nucleus transcriptomic studies of human adipose tissue, bona fide beige adipocytes remain difficult to identify and characterize *in vivo.*^21^^,^^22^ Indeed, humans live and are sampled under thermoneutral conditions that suppress thermogenic gene expression and beige adipocyte recruitment.^23^ In addition, adipose tissue biopsies are often obtained from individuals with obesity, in whom adipose tissue thermogenic capacity is markedly reduced.^24^^,^^25^ Finally, differentiating adipose progenitors constitute only a small fraction of cells in adult adipose depots,^26^ limiting the resolution of trajectory and state-mapping analyses. Therefore, controlled *in vitro* differentiation systems remain a necessary and tractable approach to resolve the molecular mechanisms governing beige vs. white adipogenesis in humans.

Thermogenic heterogeneity within human subcutaneous adipose tissue may arise from inter-individual differences in the abundance or responsiveness of progenitor cells capable of beige adipocyte differentiation. To probe this variability, we compare the thermogenic transcriptional programs of beige adipocytes derived from hASCs isolated from six unrelated healthy donors. Using scRNA-seq, we generate a high-resolution map of early adipogenesis to characterize undifferentiated hASCs and early adipogenic stages within white and beige differentiation conditions.

## Results

### Thermogenic programs in beige adipocytes are subject-specific

We have established beige and white adipose differentiation of primary hASCs isolated from the subcutaneous adipose tissue of six unrelated, normal-weight female subjects (S1–S6), in the presence or absence of rosiglitazone, respectively (Figure 1A and Table S1).^27^ When compared to beige adipocytes, the white adipocyte transcriptome is enriched for genes encoding proteins of the ECM and cell-matrix interactions, and regulators of mesenchymal stem cell development and proliferation (Figure 1B). Rosiglitazone treatment elicits a broad upregulation of mitochondrial and oxidative phosphorylation genes in beige adipocytes from all subjects (Figure 1B). Consistent with rosiglitazone’s role as a PPARγ agonist, the beige phenotype in mature adipocytes is also associated with an upregulation of adipogenesis genes, including those involved in lipid handling (Figure 1B). The expression of the beige adipocyte markers *CIDEA*, *CITED1*, *PM20D1*, and *UCP1* is upregulated across all subjects upon rosiglitazone treatment (Figure 1C). However, the extent of induction varies, with RNA-seq data indicating markedly higher *UCP1* expression in subjects S2 and S6, and significantly lower expression of *CIDEA* in subjects S1 and S2 compared to the other subjects (Figure 1C). This confirms the induction of beiging in cultured adipocytes from all six subjects and highlights differences in canonical thermogenic potential between individuals.

**Figure 1:**
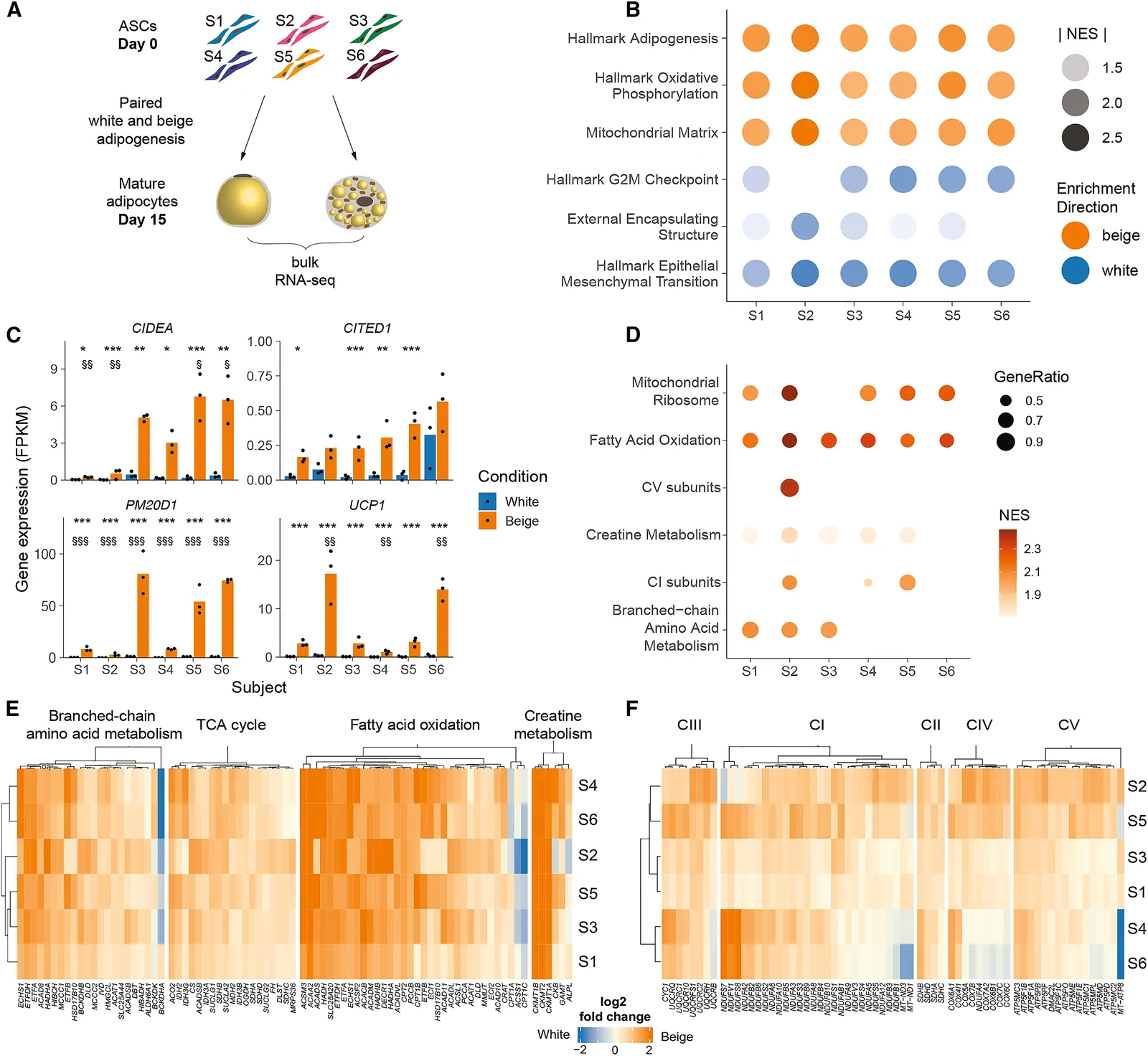
Thermogenic programs in beige adipocytes are subject-specific (A) Schematic of paired white and beige adipogenesis in hASCs from six subjects. (B) Top cellular component and Hallmark gene sets between white vs. beige adipocytes (adjusted *p* < 0.01; GSEA). (C) *CIDEA*, *CITED1*, *PDK4*, and *UCP1* expression from bulk RNA-seq of mature adipocytes from six subjects subjected to white and beige differentiation (white vs*.* beige: ∗∗∗ adjusted *p* < 0.0001, ∗∗ adjusted *p* < 0.001, ∗ adjusted *p* < 0. 01; subject vs*.* all subjects: §§ adjusted *p* < 0.0001 § adjusted *p* < 0.001; limma eBayes; *n* = 3 independent differentiation experiments). (D) Top MitoPathways (Rath et al. 2021) in white and beige adipocytes (adjusted *p* < 0.01; GSEA). (E) Heatmap of average log2 fold change (*n* = 3) for differentially expressed genes (*p* < 0.01; limma combined test) involved in “branched chain amino acid metabolism”, “TCA cycle”, “fatty acid metabolism” (MitoPathways; some genes belong in multiple pathways), and creatine metabolism genes. (F) Heatmap of average log2 fold change (*n* = 3) for differentially expressed subunits of the five oxidative phosphorylation complexes CI – V (*p* < 0.01; limma combined test). NES, normalized enrichment score; GeneRatio: the fraction of genes in the gene set contributing to the score.

To further characterize potential subject-specific thermogenic phenotypes, we conducted a gene set enrichment analysis (GSEA) of beige vs. white adipocytes on MitoPathways (MitoCarta^28^). All subjects display a beige-specific increase in the expression of fatty acid oxidation genes (Figure 1D). Interestingly, other alternative thermogenic pathways show differential enrichment between subjects. For instance, branched-chain amino acid metabolism is significantly enriched in only three subjects (Figures 1D and S1–S3). Conversely, *CKB* and *ALPL*, which facilitate futile creatine cycling in thermogenic adipocytes,^29^ are strongly upregulated in the beige adipocytes from subjects S1, S4, and S6 (Figure 1E). This suggests that fatty acid oxidation is a widespread thermogenic mechanism in subcutaneous beige adipocytes, while activation of other alternative thermogenic pathways, such as branched chain amino acid metabolism and creatine cycling, is individual-specific.

Similarly, genes encoding subunits of the oxidative phosphorylation complexes, reported as markers of UCP1-negative thermogenic adipocytes,^30^ are differentially enriched between subjects. Of note, subject S2 has the highest beige-specific upregulation of complex V, and a unique pattern of subunit expression for complexes I, III, and IV compared to other subjects (Figure 1F). For example, complex I subunits *NDUFS7* and *NDUFS8* are enriched in white adipocytes in subject S2, but are strongly beige-specific in subjects S4–S6. Despite an overall upregulation of mitochondrial genes in beige adipocytes, mitochondria-encoded genes such MT-*ND3*, MT-*ND1*, and MT-*ATP8* are enriched in white adipocytes among several subjects, particularly S4 and S6. Thus, the transcriptional regulation of mitochondrial energy programs varies between individuals. Overall, rosiglitazone induces beiging in six subjects and activates subject-specific thermogenic programs that include differential expression of *UCP1*, oxidative phosphorylation complexes, and enzymes involved in non-canonical thermogenic pathways.

### Adipose progenitors display subject-specific transcriptomes

To explore whether baseline transcriptional differences might underlie these subject-specific thermogenic programs, we profiled progenitor populations prior to adipogenic induction. We performed scRNA-seq on confluent, non-induced progenitor cells (day 0) followed by unsupervised clustering and uniform manifold approximation and projection (UMAP) visualization on approximately 8,000 cells per subject. Most progenitors from subjects 1, 5, and 6 occupy overlapping territories in the UMAP space and share clusters, indicating high transcriptomic similarity even before correcting for inter-sample variability (Figures 2A and 2B). Conversely, subjects 2, 3, and 4 occupy separate UMAP territories from the other subjects, highlighting differences in their transcriptomes. Indeed, we observe that strong transcriptional signatures, such as cell cycle gene expression, result in cluster overlap between subjects (Figures 2C–2E, S1A, and S1B; cluster u7). Interestingly, another shared cluster is enriched for *CD36*, *TINAGL1*, and actin cytoskeleton genes (Figures 2C–2E and S1; cluster u8), previously reported as markers for cells with high adipogenic potential.^31^

**Figure 2:**
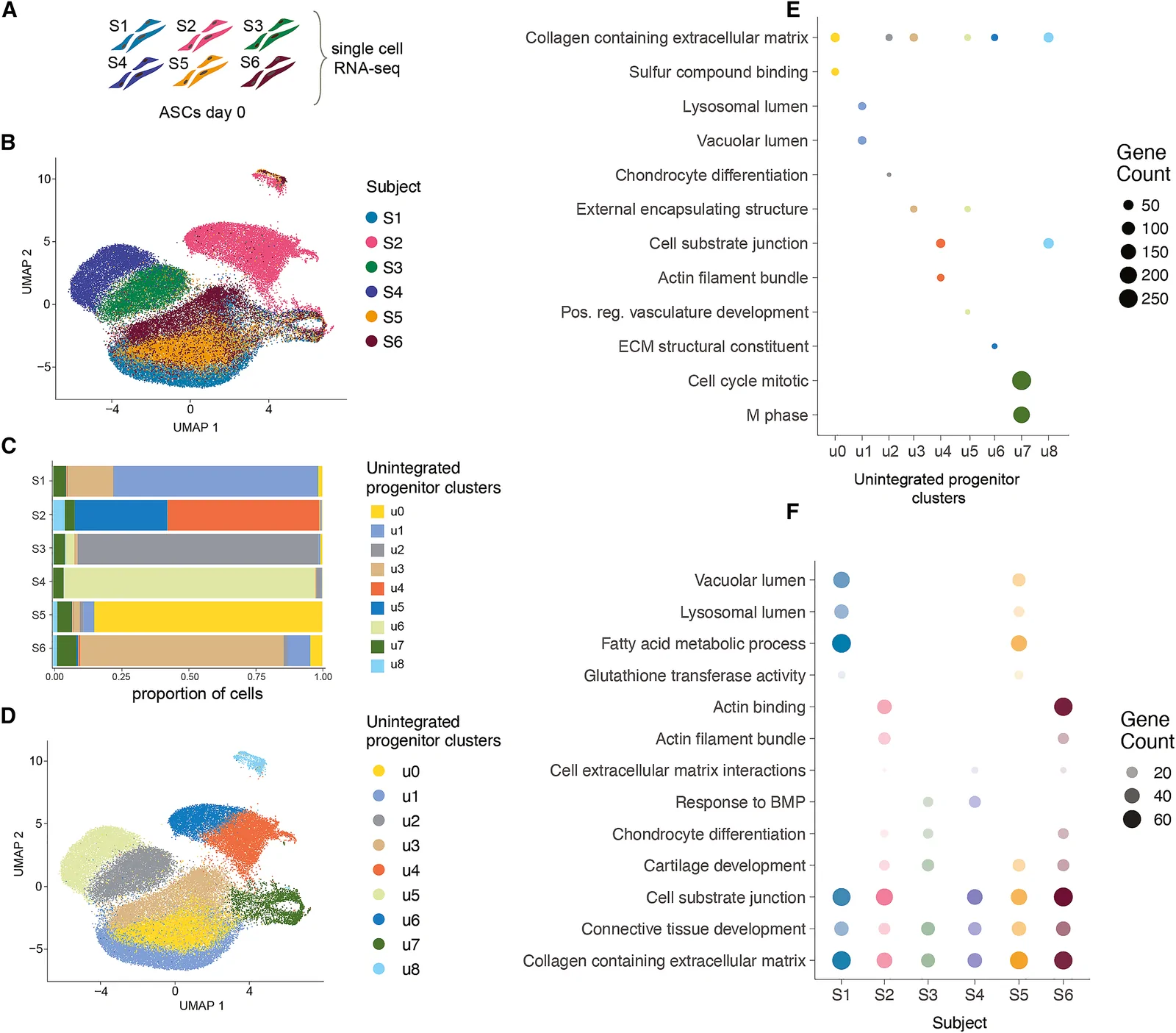
Adipose progenitors display subject-specific transcriptomes by scRNA-seq (A) Overview of progenitor scRNA-seq in six subjects (S1–S6). (B) UMAP of 56,638 progenitor cells colored by subject. (C) Proportion of cells from each subject assigned to clusters. (D) UMAP colored by unintegrated clusters (u0–u8; Louvain clustering, resolution 0.5). (E and F) Enriched gene sets in cluster marker genes (E) and between subjects (F) (adjusted *p* < 0.01 in overrepresentation test of differentially expressed genes [adjusted *p* < 0.01, Wilcoxon test, cells as replicates]).

To identify transcriptome features associated with each subject, differential expression and GSEA were conducted between subjects (Figure 2F). Strikingly, genes related to ECM and connective tissue development are differentially expressed in all subjects, indicating unique modulation of the extracellular environment (Figure 2F and Table S2). We also detect subject-specific enrichment for fatty acid metabolism (S1 and S5) and bone morphogenic protein (BMP) response (S3 and S4) terms. Notably, cells from subjects S2 and S6, which show the highest induction of *UCP1* expression in beige adipocytes (Figure 1C), also display a global enrichment for actin-binding genes at the progenitor stage (Figure 2F). Thus, prior to data integration, hASCs from different subjects share a cell cycle cluster, while differential expression of genes related to ECM, fatty acid metabolism, and actin binding drives subject-specific clustering.

### Harmonization reveals subject-specific shifts in progenitor cell states

To identify gene expression patterns conserved across subjects, we next integrated the scRNA-seq datasets, testing four unsupervised methods for removing subject-specific variation: RPCA (Reciprocal Principal Component Analysis),^32^ Harmony,^33^ FastMNN (Mutual Nearest Neighbors)^34^ and ScVI (Single cell Variation Inference)^35^ (Figure S2; see STAR Methods). All four methods aggregate most cells in a central group, except for the cell cycle and *CD36* cell clusters, which consistently separate in the UMAP space and are shared between subjects. Since RPCA integrated clusters initially annotated to more specific ontology terms, this method was selected for further optimization of clustering resolution (Figure S3), resulting in seven integrated progenitor clusters (Figure 3A; p0-p6).

**Figure 3:**
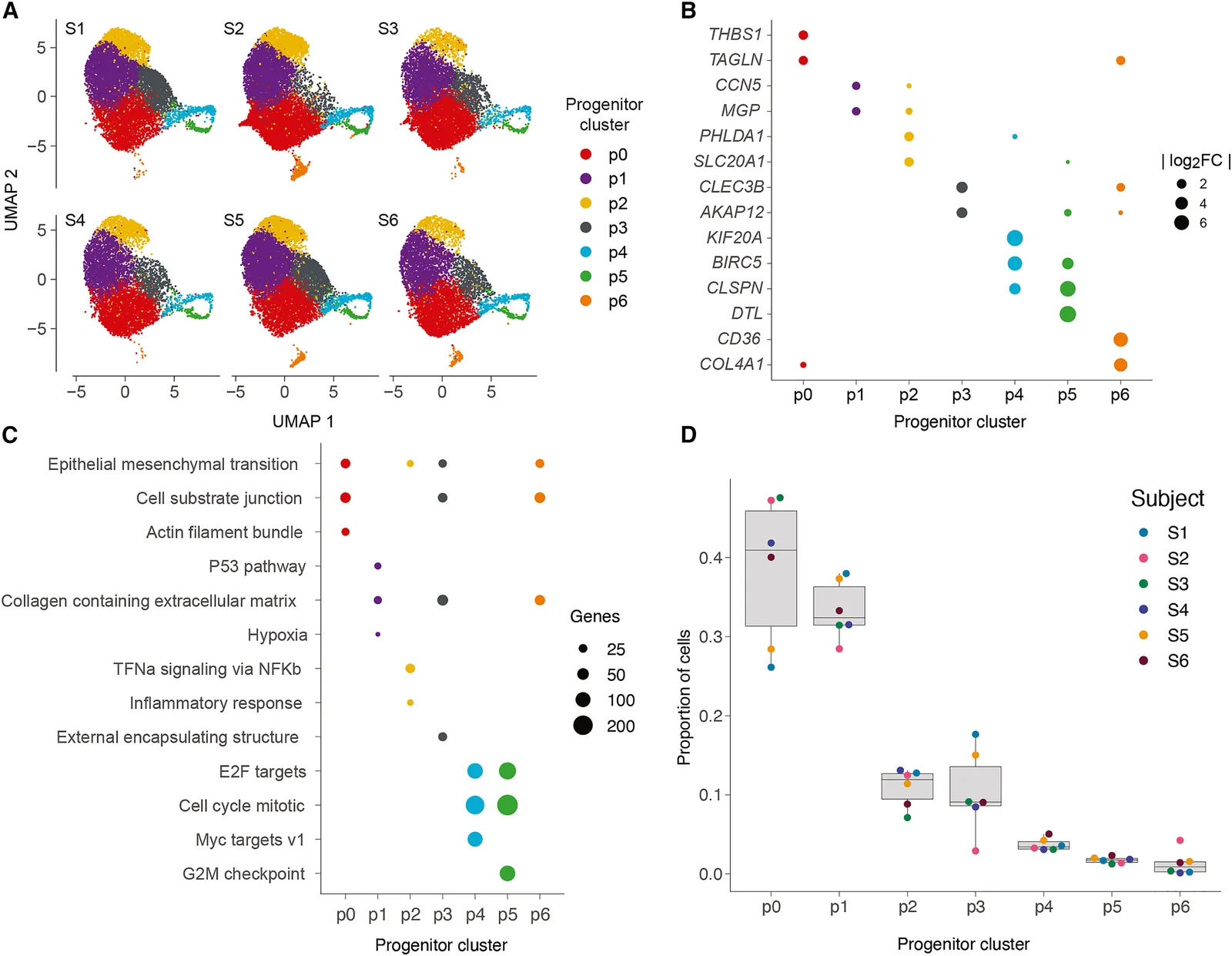
Harmonization of scRNA-seq reveals subject-specific shifts in progenitor cell states (A) UMAP of cells from progenitor scRNA-seq colored by progenitor clusters (p0–p6; Louvain clustering, resolution 0.3, 56,638 cells). (B) Dot plot of top marker genes in clusters (false discovery rate [FDR] adjusted *p* < 0.01 & log2 FC > 0.5; Wilcoxon test, cells as replicates). (C) Top GO terms enriched in each cluster (adjusted *p* < 0.01; Fisher’s exact). (D) Proportion of cells per subject assigned to each cluster.

Integrated clusters describe cells enriched for cell cycle progression and components of the ECM, cytoskeleton, and cell-cell junctions (Figures 3A–3C). Indeed, the first UMAP dimension displays a strong gradient separating cell cycle clusters (p4 and p5) from the p53 cluster (p1) (Figures 3A–3C and S4A–S4D). The second UMAP dimension describes opposing clusters enriched for ECM genes (e.g., *MGP*, *CLEC3B*; clusters p1 and p3) vs. actin binding genes (e.g., *TAGLN*; cluster p0). The TNF-α signaling cluster p2 is enriched for NF-κβ target genes, including *ICAM1*, *CXCL6* and *CXCL8* (Tables S3 and S4). Consistent with prior reports,^13^ *DPP4* expression is enriched in cluster p3, which lies adjacent to proliferative clusters, whereas *F3* shows modest enrichment in p0 (Figures S4A–S4D). In contrast, other markers previously associated with thermogenic potential are either not detected (*ALPL*^36^) or broadly expressed across progenitor clusters, with a relative enrichment in cluster p3 and p6 (*CD81*^11^).

Data integration highlights variable proportions of progenitor clusters between subjects. In particular, the proportion of cells in clusters related to cytoskeleton (p0) and ECM (p1 and p3) are highly variable (Figure 3D), consistent with our unintegrated data analysis (Figure 2F). Of all the subjects, S2 has the most distinct cluster composition, with a higher representation of the actin cytoskeleton clusters (p0) and a lower representation of the ECM clusters (p1 and p3). Conversely, subjects 1 and 5 are depleted for the actin cytoskeleton cluster (p0) compared to the other subjects. Overall, we find a significant variation in populations of subcutaneous hASCs derived from the adipose tissue of different individuals, driven by differential expression of genes related to the ECM, actin, and cell-cell signaling.

### Dynamic cell state transitions in early adipogenesis

To test how these different adipose progenitor populations respond to adipogenic induction, we extended our analysis into early adipogenesis. Subject S6 hASCs, which have been extensively characterized in our previous work, including detailed phenotypic and transcriptional analyses of white and beige adipocyte differentiation,^37^ display the widest range of progenitor transcriptional signatures and a strong beiging potential (Figures 1C and 3D). This hASC population was therefore selected for scRNA-seq 1 and 3 days after induction of white adipose differentiation.

By day 3, the adipogenic program is fully initiated^38^: key adipogenic markers are expressed and cells start accumulating lipid droplets (Figures S5A–S5D). Consistent with this commitment, progenitor (day 0) cells clearly separate from differentiating cells in the UMAP, while day 1 and day 3 cells are contiguous (Figures 4A and S6A), reflecting the pronounced effect of adipogenic induction media on hASC states. To directly compare our *in vitro* differentiating adipose progenitors with *in vivo* human datasets, we performed label transfer using two independent single-cell atlases of human subcutaneous adipose tissue^16^^,^^22^ (Figures 4B and 4C). Day 0 cells predominantly map to ASPC2 population from Emont et al., (Figure 4B) whereas label transfer from Lazarescu et al. assigns cells to ASCP3 population (Figure 4C), a progenitor subset enriched for fibroadipogenic features. By day 1, differentiating cells displayed increased heterogeneity, with assignments distributed across multiple *in vivo* ASPC populations while a substantial fraction remains unassigned (Figures 4B and 4C). Notably, most cells transitioning toward adipogenesis at day 3 are not detected in single-nuclei RNA-seq analyses of whole adipose tissue. This likely reflects the limited capture of early differentiating cells in human adipose tissue datasets, and underscores the enhanced temporal and cellular resolution achieved by our *in vitro* system.

**Figure 4:**
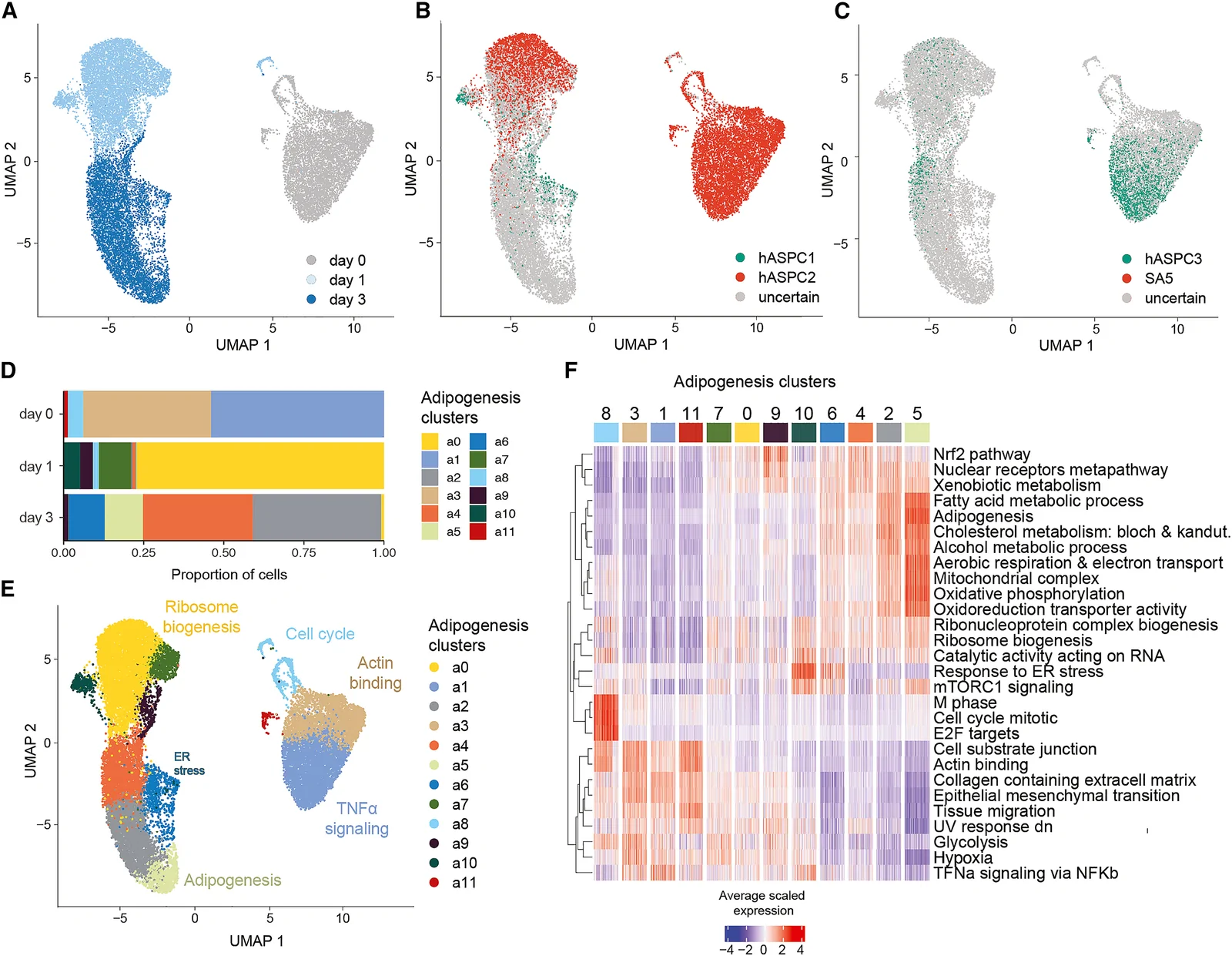
scRNA-seq resolves cell states contributing to early adipogenesis (A) UMAP of 21,794 cells sampled from uninduced (day 0) and induced hASCs (day 1 and day 3) for scRNA-seq, colored by sampling time. (B and C) Labeling of adipogenic cells using previously defined adipose populations from human biopsies with Azimuth, mapping to the reference atlas from (B) Emont et al.^16^ or (C) Lazarescu et al.^22^ (D) Proportion of cells from each time point in clusters (Louvain clustering, resolution 0.5). (E) UMAP colored by adipogenesis clusters a0–a11. (F) Average gene expression of the top 3 GO terms per cluster. Subset of 100 cells shown per cluster.

Each differentiation time point is categorized into four main clusters (adipogenesis clusters a0 to a11; Figure 4D), recapitulating sequential transcriptional changes occurring during adipogenic induction (Figure 4E). Day 0 clusters (a1, a3, a8, and a11) collectively display high expression of mesenchymal markers, ECM- and actin-related genes, which gradually decrease in day 1 and day 3 clusters (Figures 4E and S6B and Table S5). Indeed, the proportion of cells expressing cell cycle markers is drastically reduced by day 1 (cluster a8), and no cell cycle cluster is found on day 3. Instead, day 3 clusters (a2, a4, and a5) display a gradual upregulation of adipogenesis genes related to fatty acid metabolism, mitochondrial matrix, and oxidative phosphorylation, culminating in a robust induction of the adipogenic program in cluster a5. Strikingly, both day 1 cluster a8 and day 3 cluster a6 are specifically enriched in endoplasmic reticulum (ER) stress response genes, and their UMAP localization suggests that these cells form a parallel differentiation trajectory. Thus, we describe a range of cell states activated upon adipogenic induction, with early remodeling of ECM gene expression developing into an upregulation of adipogenesis, fatty acid metabolism, and mitochondrial genes by day 3.

### White- and beige-induced cell populations overlap in early adipogenesis

To address whether beige adipocytes might derive from specific progenitors, we next examined the early stages of white vs. beige adipogenic induction (Figure 5A). Prior to integration, white- and beige-induced cells largely overlap in the UMAP space (Figures S7A–S7E), although the ER stress cluster b5 shows a slight increase in the proportion of beige cells (Figures 5B, S7B, and S7D). Clustering of white and beige scRNA-seq datasets identifies an additional day 3 cluster (b10) adjacent to the day 1 cells in UMAP, that expresses mesenchymal markers and likely represents SWAT cells^18^^,^^19^ (Figures S7A–S7D). However, we find no difference in the proportion of white and beige cells within adipogenesis clusters (b1, b2, and b4) and overall, a similar activation of adipogenesis between the conditions in all clusters (Figures 5B and S7E). Only a small number of genes are differentially expressed between white and beige adipogenesis at day 1 and day 3 (Figure S7E), many of which are mitochondria-encoded transcripts. This might indicate an early remodeling of mitochondrial transcription elicited by rosiglitazone, since the mitochondrial genes MT-*ND1*, MT-*ND3*, and MT-*ATP8* are white-specific in the mature adipocytes of this subject (Figure 1F). However, mitochondrially encoded transcripts commonly contribute noise to scRNA-seq, and we find no differentially expressed mitochondrially encoded genes between white and beige cells in bulk RNA-seq of a parallel early differentiation time course (Figure S7F).

**Figure 5:**
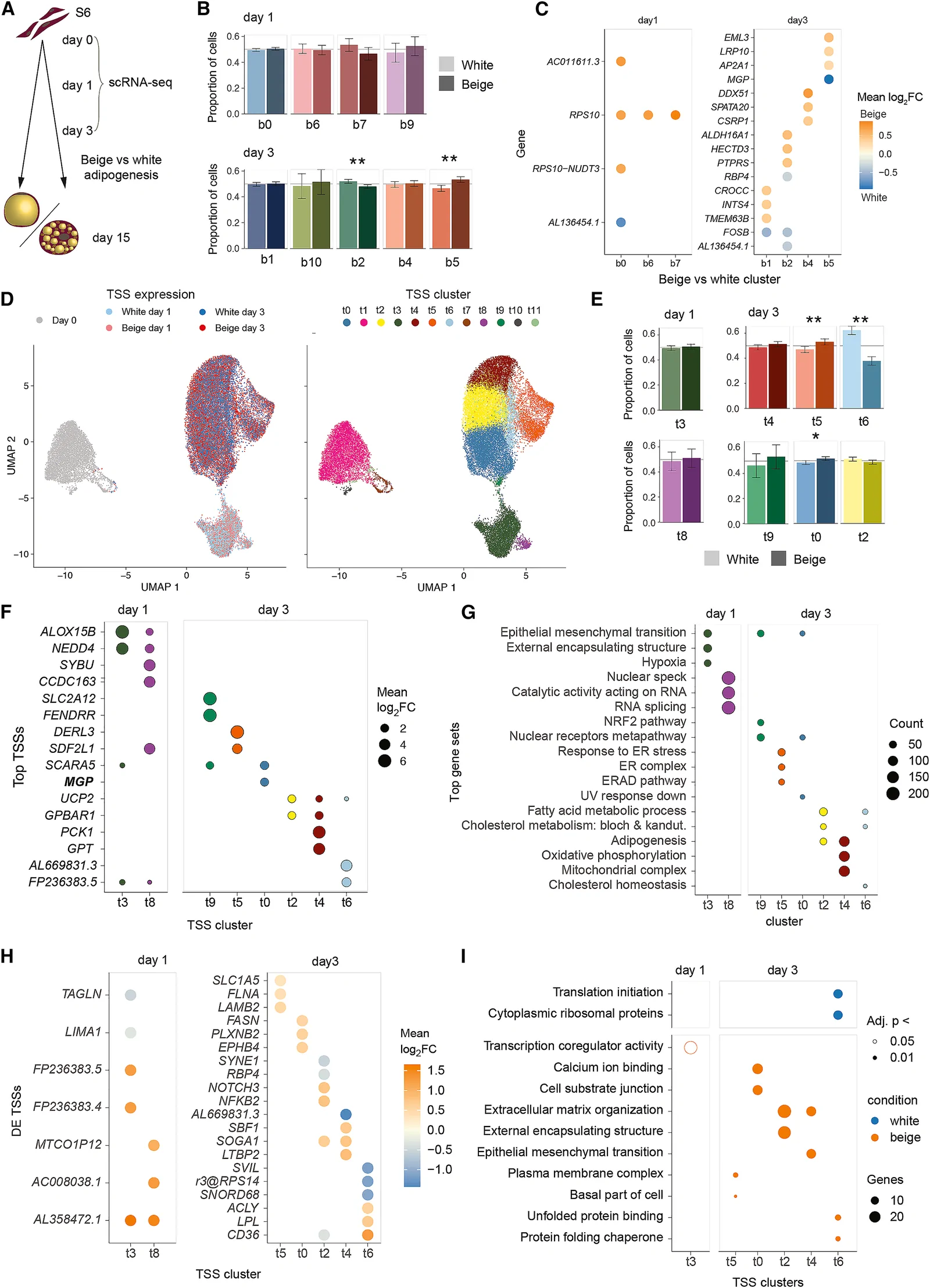
TSS regulation in early adipogenesis of beige-treated progenitors (A) Diagram of our experimental setup; scRNA-seq in early beige and white adipogenesis (day 0, day 1, and day 3) in one subject. (B) Proportion of cells from each condition in each beige vs. white cluster (b0–b12; ∗∗ FDR adjusted *p* < 0.01, chi-squared test, error bars show 99% confidence interval [CI]). (C) Dot plot of average log2 fold change (FC) for cluster-specific differentially expressed genes between white and beige cells (clusters b0–b11). (D) TSS-level UMAP colored by condition/time (left) and TSS clusters (t0–t12; right images; day 0 = 7,309 cells, day 1 = 2,459 down sampled white cells and 2 459 beige cells, day3 = 10 848 white and 10 848 down sampled beige cells). (E) Proportion of white and beige cells per TSS cluster (∗ FDR adjusted *p* < 0.05, ∗∗*p* < 0.01, chi-squared test, error bars show 99% CI). (F) Dot plot of top TSSs in clusters (adjusted *p* < 0.01; Wilcoxon test, cells as replicates). (G) Gene Ontology (GO) terms per TSS cluster. (H) Dot plot of average log2 FC for top differentially expressed annotated TSS (shown as gene name) between white and beige cells in each TSS cluster (t0–11; adjusted *p* < 0.01 & |log2 FC| > 0.25, Wilcoxon test). Mitochondrially encoded genes and TSSs not shown). (I) GO terms enriched between white and beige cells in each TSS cluster (t0–t11; adjusted *p* < 0.05 or *p* < 0.01 as indicated and number of genes >2, Fisher’s exact test).

We next compared gene expression within each beige vs*.* white cluster (b0–b12; Figure S7G). At day 1, beige cells show enrichment for ribosomal protein genes, including *RPS10* (Figure 5C and Table S6). By day 3, differentially expressed genes span a broader range of biological functions. For example, beige cells have higher expression of the ribosomal biogenesis enzyme *DDX51* as well as structural components such as *EML3*, *CSRP1*, and *CROCC*. Concordant with previously reported signatures of AP1 transcription factors in mature white vs. beige adipocytes,^37^ *FOSB* transcripts are enriched in white cells within early adipogenic clusters (b1 and b2). Although more differentially expressed genes are detected within clusters, the log_2_ fold changes are modest, confirming that the gene-level beige and white transcriptomes are similar in early adipogenesis.

### Subtle shifts in the TSS-level transcriptome affect extracellular matrix and lipid metabolism genes in beige-induced progenitors

As many beige-specific genes display alternative transcription start site (TSS) usage in mature adipocytes,^27^^,^^39^ we reanalyzed our scRNA-seq dataset with SCAFE,^40^ which uses reads from the 5′ end of transcripts to estimate expression at the TSS-level. TSS-level scRNA-seq analysis of beige and white cells leads to similar clusters (t0-t11; Figure 5D) as gene-level analysis. To validate our approach, we examined the expression of *PPARγ* isoforms along the adipogenic trajectory. As expected, *PPARγ*1 expression increases from day 1, while *PPARγ*2 is induced in day 3 late adipogenic clusters, and readily detected at the protein level at this differentiation time point (Figure S8).

In contrast to the gene-level analysis, we find different proportions of white and beige cells within day 3 TSS clusters, with the SWAT cluster t7 being enriched, and the cholesterol metabolism cluster t8 depleted for beige cells (Figures 5E–5G and S9A–S9C). TSS-level expression analysis reveals an increased expression of *COL6A2* in beige cells at day 3 (Figure S9D), consistent with a higher proportion of SWAT cells. Low Density Lipoprotein receptor-related protein 1 (*LRP1*) and fatty acid synthase (*FASN*) TSSs are also beige-enriched (Figure S9D), potentially contributing to decreased cholesterol metabolism.^41^ This indicates that early shifts in TSS-level transcription associated with beige differentiation, relative to white differentiation, are related to ECM composition, and cholesterol and lipid metabolism.

Comparing TSS expression within clusters, we find a significant enrichment for genes related to transcription coregulator activity (GO:0003712) in the beige cells at day 1 (Figures 5H and 5I; cluster t3; adj. *p* = 0.0194), suggesting that early differences in white and beige trajectories may begin at the level of chromatin modification and transcription regulators. In day 3 clusters, beige-upregulated TSSs annotate to ECM-related Gene Ontology (GO) terms. Differentiating beige cells (clusters t0, t1) show higher expression of the glycoprotein *EMILIN1*, the actin-glycoprotein crosslinker *FLNA* and the surface receptor and mechanosensor *PLXND1* (Figure 5H and Table S7). Further, beige cells from SWAT cluster t7 show higher expression of *LAMC1* and *PHLDB1* (Figures 5H and 5I), suggesting that beige adipogenic induction also results in differential ECM remodeling by multipotent cells. In contrast, white-induced cells from day 1 cluster t3 display higher expression of actin-binding genes *LIMA1* and *TAGLN*. This reiterates that fate-specific remodeling of the ECM and cytoskeleton are key components of the white vs. beige trajectory in early adipogenesis.

Within the day 3 cholesterol metabolism TSS cluster (t8), which contains a higher proportion of white cells (Figures 5D–5F and S9C), the actin regulator SVIL (isoform 1) and ribosomal protein genes are upregulated in white-induced cells (Figures 5G and 5H), while beige-induced cells express higher levels of *LPL*, *ACLY*, *FASN*, and protein folding chaperones. Thus, within this shared metabolic cluster, beige induction elicits an upregulation of key *de novo* lipogenesis enzymes. Overall, TSS-level analysis of unintegrated scRNA-seq data detects differential expression during beige and white adipogenesis. This involves subtle upregulations of ECM and lipid metabolism genes in beige-induced cells, but not a distinct cell state at this early stage of adipogenesis.

### Trajectory analysis of beige and white adipogenesis identifies a cluster of committed progenitors

To compare white and beige trajectories during early adipogenesis, we evaluated three different integration strategies for their ability to infer continuous temporal transitions (Figure S10). Integration with Harmony separates day 0 cells from differentiating cells, while both FastMNN and RPCA connect all three time points together (Figure S10). RPCA achieves the highest degree of integration, overlapping all three time points, thus masking temporal progression. In contrast, FastMNN generates a continuous trajectory spanning day 0 to day 3, while preserving temporal structure. We therefore selected FastMNN as the integration strategy best suited to resolve adipose stem cell progression through adipogenesis.

Following joint integration of white and beige datasets with FastMNN, we predicted white and beige trajectories separately using Monocle3. In both time courses, a main adipogenic trajectory converging on cluster t4 is detected (Figure 6A). A secondary branch captures the sparsely populated ER stress and cholesterol metabolism clusters (t8, t5, and t6). We next estimated adipogenic pseudotime scores separately for the beige and white trajectories, fixing the center of cluster t4 as the endpoint. Day 0 clusters (t7, t11, t1, and t10) map to early pseudotime (Figure 6B), whereas most day1 cells (t3) are assigned intermediate pseudotime values, similar to the day3 SWAT cluster (t9). The remaining adipogenesis clusters progress to later pseudotime, from t0 to t2 and ultimately t4, accompanied by increasing expression of adipogenic genes. Comparison of pseudotime distributions between white and beige cells within each TSS cluster reveals highly concordant progression (Figure 6B), with preserved cluster ordering and overlapping pseudotime distributions. Concordant results are obtained when the analysis is performed at the gene level (Figures S11A and S11B), indicating that differentiation kinetics are largely shared between the two conditions.

**Figure 6:**
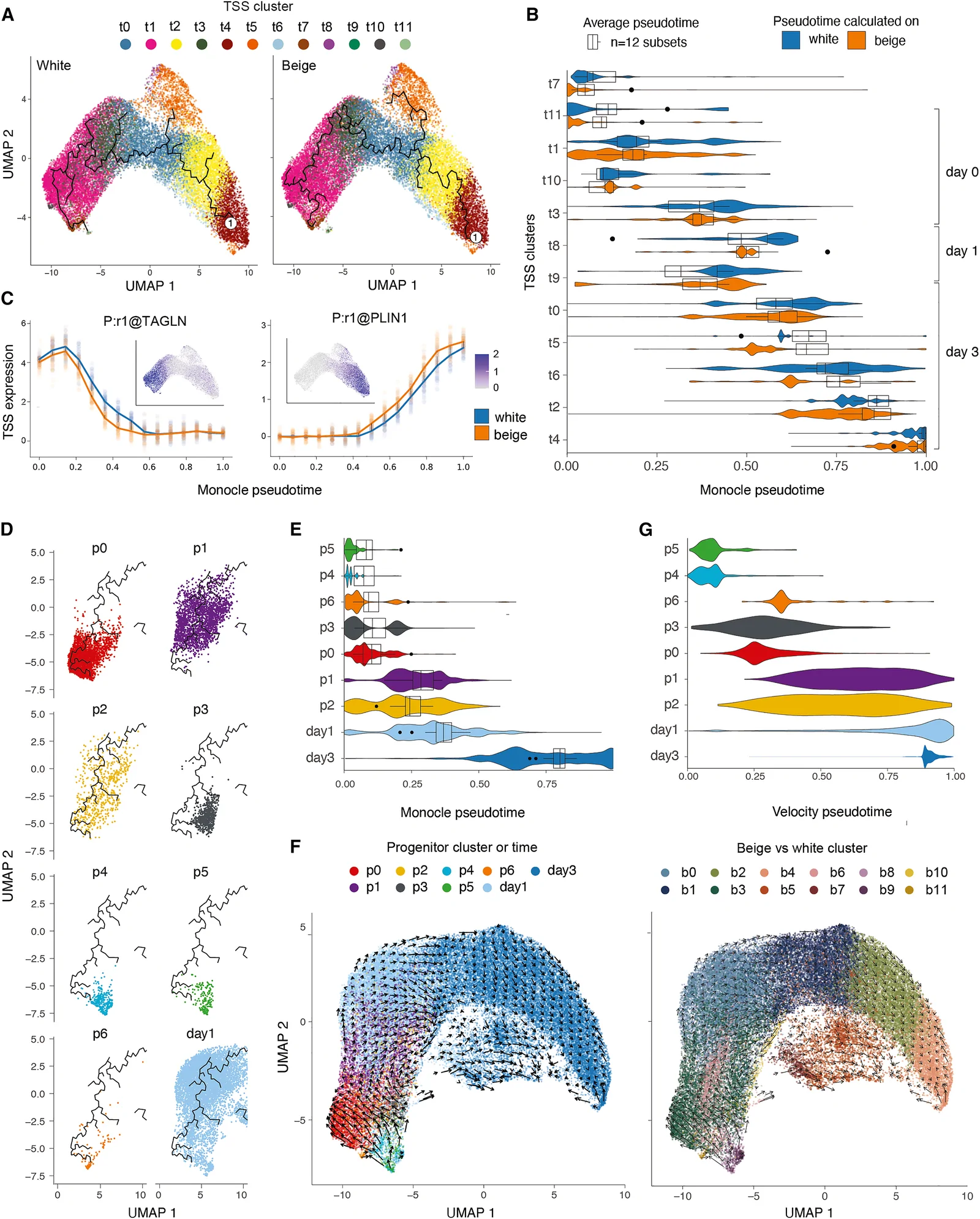
Trajectory analysis of beige and white adipogenesis identifies committed progenitors (A) Monocle3 trajectory graph of white or beige adipogenesis integrated at the TSS-level with FastMNN (*n* = 7,309 day 0 cells, 2,459 day 1 cells, and 10,848 day 3 cells for each trajectory [white and beige]). (B) Pseudotime calculated for white and beige differentiation trajectories (Monocle3 on FastMNN UMAPs). Violin plots show cells within each TSS cluster; boxplots with outliers show the average pseudotime for each of 12 trajectory simulations on cell subsets. (C) Alignment of beige and white pseudotime trajectories for TSSes of TAGLN and PLIN1 (Genes2Genes). Insets show the distribution of single cell TSS expression across the UMAP. (D) Zoom on early trajectory (day 0/day 1), split by progenitor cluster and day 1 (*n* = 7,265 day 0 cells, 7,637 day 1 cells, and 11,077 day 3 cells [not shown]). (E) Monocle3 pseudotime densities grouped by progenitor clusters (day 0), and later time points (day 1 and day 3); boxplots represent average pseudotime for 12 trajectory simulations (white and beige gene-level). (F) Grid-based RNA velocity arrows (scvelo) overlaying 44,693 cells colored by progenitor cluster (left) or white vs. beige clusters (right). (G) Velocity pseudotime densities grouped by progenitor clusters (day 0), and later time points (day 1 and day 3).

To directly compare transcriptional dynamics along white and beige differentiation, we performed trajectory alignment across pseudotime using Genes2Genes.^42^ On average, Genes2Genes reports complete similarity between white and beige trajectories at both the TSS and gene levels (Figure S11C and Tables S8 and S9). Nonetheless, a subset of TSSs and genes displays some variation in expression dynamics: higher expression of the TAGLN TSS in the first half of the white trajectory matches its differential expression at day1 (Figure 6C, see also Figure 5H), whereas perilipin1 (PLIN1) TSS expression is enriched in the latter half of the beige trajectory (Figure 6C). Notably, this divergence is confined to specific TSSs and is not observed at the gene level (Figure S11D), indicating promoter or isoform-specific regulation. This aligns with our previous findings supporting the functional relevance of isoform-level regulation in the establishment of beige vs. white identity.^27^

The large concordance between white and beige adipogenic trajectories prompted us to ask whether both phenotypes arise from the same progenitor states. To assess the relative commitment of progenitor populations, we annotated day 0 cells with their progenitor cluster identities (Figures 3 and S11A). Proliferating clusters p4 and p5 map to the earliest predicted pseudotime, consistent with proliferation opposing adipogenic commitment^43^ (Figures 6D and 6E). The largest progenitor cluster, p0, together with p3 and p6, corresponds to intermediate pseudotime and defines the early day 0 trajectory (Figures 6D and 6E). In contrast, clusters p1 and p2 overlap extensively with day 1 cells in both pseudotime and UMAP space, indicating transcriptional profiles aligned with early differentiating cells, and suggesting that these clusters represent the most committed progenitor populations.

To validate these findings, we applied RNA velocity, an unsupervised approach that estimates the direction of transcriptional changes in a latent space and, using a dynamical model, predicts pseudotime without user-defined start or endpoints. Gene-level RNA velocity analysis on integrated UMAPs identifies two trajectory endpoints corresponding to the two branches of adipogenesis^18^: one at the interface of all three time points, within the SWAT cluster (b10), and another in day 3 cells undergoing adipogenesis (clusters b2 and b4) (Figures 6F and S11A). Velocity vectors originate from proliferating clusters p4 and p5 and trace a temporal progression consistent with Monocle3 pseudotime reconstruction (Figures 6F and 6G). Together, these orthogonal approaches position p1 and p2 at the transition toward adipogenic commitment along a shared white and beige differentiation trajectory, supporting the possibility of a common progenitor origin.

### ICAM1-positive progenitors display dual white and beige potential

To test whether white and beige adipocytes can arise from a shared progenitor, we isolated a defined progenitor subset and assessed its white and beige differentiation potential. We focused on cluster p2 because it: (1) expressed the surface protein ICAM1 (Figures 7A and 7B), enabling FACS-based isolation, (2) forms a highly homogenous cluster that is stable across clustering resolutions (Figure S3A), (3) represents a small fraction of day 0 cells (∼10%; Figure 3D), (4) displays robust adipogenic potential based on prior data,^13^ and (5) specifically expresses *CXCL8* (Figures 7A and 7B), a reported marker of inducible beige progenitors.^44^ We enriched cluster p2 hASCs by selecting ICAM-high cell (top 10%; ICAM1^hi^) using fluorescence-activated cell sorting (FACS) (Figures 7C, 7D, and S12). As expected, ICAM1^hi^ cells show elevated *CXCL8* expression (Figure 7D), confirming successful enrichment of cluster p2 subpopulation.

**Figure 7:**
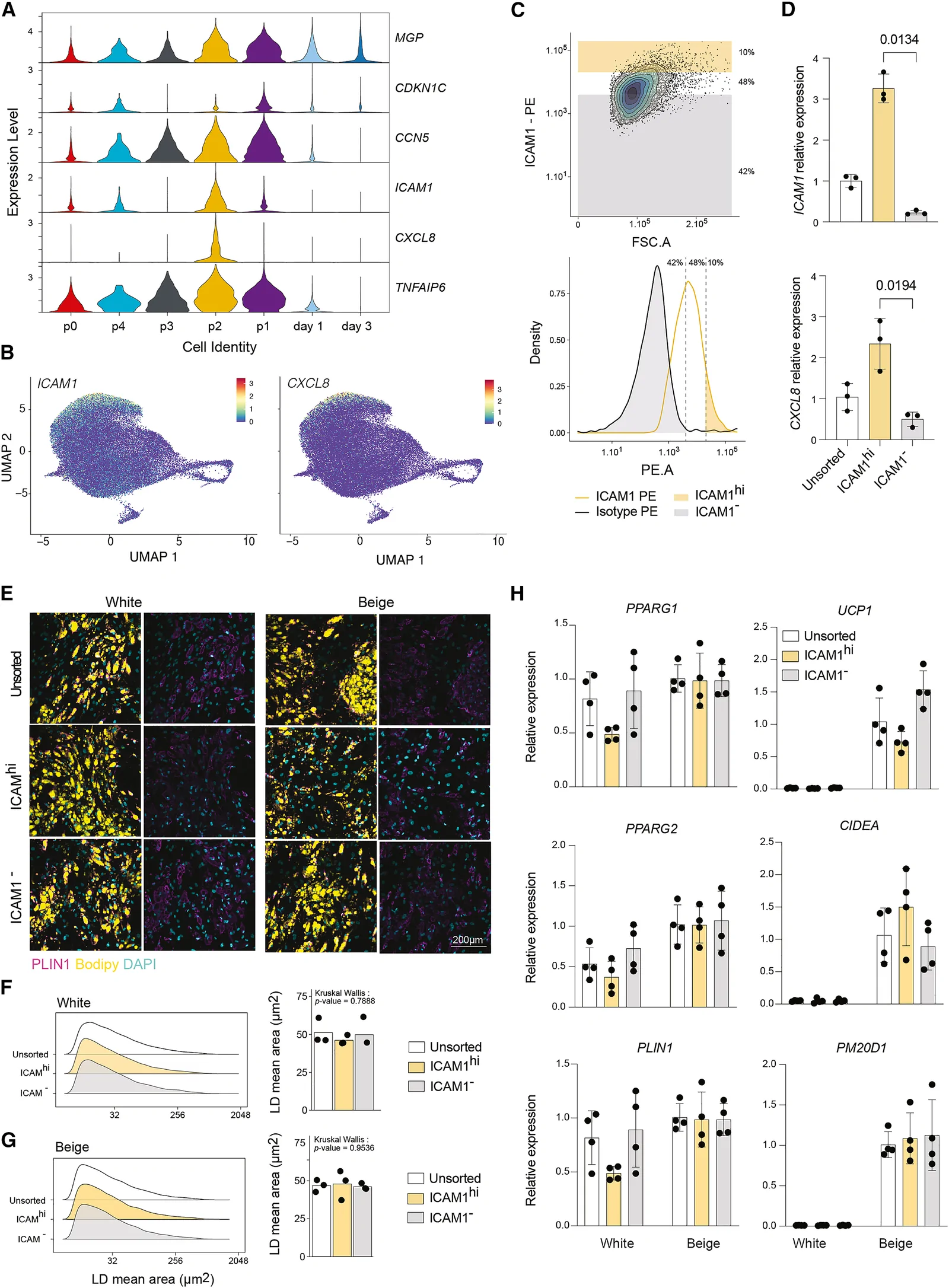
ICAM^hi^ progenitors display dual white and beige adipogenic potential (A) Violin plots of key p2 marker gene expression across selected progenitor clusters and cells at day 1 and day 3. (B) Expression of p2 markers *ICAM1* and *CXCL8* on RPCA-integrated progenitor UMAPs. (C) FACS scatterplot of ICAM1-PE vs. FSC-A (top) and density plot of ICAM1-PE and isotype-PE (bottom) in day 0 hASCs. Gates define ICAM1^hi^ (10%) and ICAM1^-^ (42%) populations (representative plots from *n* = 4 independent sorting experiments). (D) Quantitative reverse-transcription PCR (RT-qPCR) analysis of *ICAM1* and *CXCL8* expression in unsorted, ICAM1^hi^ and ICAM1^-^ sorted populations (mean with SD; Kruskal-Wallis test, *n* = 3 independent sorting experiments). (E) Immunostaining of perilipin1 (PLIN1) and bodipy and DAPI stainings in day 15 white and beige adipocytes differentiated from unsorted, ICAM1^hi^ and ICAM1^-^ sorted populations (representative images from *n* = 3 fields per condition and per replicate, across 3 independent differentiation experiments; scale bar 200 μm). (F and G) Distribution of lipid droplet area (left) and mean lipid droplet area in day 15 white (F) and beige (G) adipocytes differentiated from unsorted, ICAM1^hi^ and ICAM1^-^ sorted populations (Kruskal-Wallis test, > 80,000 lipid droplets quantified in *n* = 3 independent differentiation experiments). (H) RT-qPCR analysis of adipogenesis markers (*PPARG1*, *PPARG2*, and *PLIN1*) and beige adipocyte markers (*UCP1*, *CIDEA*, and *PM20D1*) in day 15 white and beige adipocytes differentiated from unsorted, ICAM1^hi^ and ICAM1^-^ sorted populations (mean with SD; Kruskal-Wallis test, *n* = 4 independent differentiation experiments).

We next differentiated ICAM1^hi^, ICAM1^-^, and unsorted hASC into white and beige adipocytes. At day 15, lipid droplets staining shows that the differentiation efficiency of ICAM1^hi^ cells is comparable to that of the unsorted population under both white and beige differentiation conditions (Figure 7E), with no significant difference in lipid droplet size (Figures 7F and 7G). Expression levels of *PPARG1*, *PPARG2*, and *PLIN1* further confirm the robust white adipogenic capacity of ICAM1^hi^ cells (Figure 7H), consistent with previous reports.^13^ Strikingly, under beige differentiation conditions, ICAM1^hi^ cells express *UCP1*, *CIDEA*, and *PM20D1* at levels indistinguishable from those of the unsorted population (Figure 7H). Together, these data demonstrate that ICAM1^hi^ hASCs retain full white and beige adipogenic competence and thus represent a human adipose progenitor population with dual white and beige potential.

## Discussion

We investigated variation in the gene expression profile of progenitor populations of subcutaneous hASCs derived from the adipose tissue of six unrelated individuals, and the diversity of thermogenic mechanisms elicited during beige adipogenesis in these subjects. By examining adipogenesis at the single-cell level with a focus on the early commitment phase, we characterize a range of cell states successively activated upon white and beige adipogenic induction. Our trajectory analyses identify a pool of progenitors primed toward adipogenic commitment, from which both white and beige adipocytes can emerge.

We characterize adipose progenitor populations in controlled conditions, highlighting differences in the expression of genes from the cytoskeleton, ECM, and TNF-α signaling pathways. These populations are consistent with progenitors described *in vivo*,^16^^,^^17^ indicating that the heterogeneity of the adipose stem cell progenitor pool is maintained *in vitro* even after several passages. This suggests that progenitor identity is, at least in part, maintained by intrinsic transcriptional and/or epigenetic programs. We identified cell clusters with high expression of p53 pathway and TNF-α signaling pathway-related genes as the most committed to adipogenesis. Indeed, our trajectory analyses describe a continuum, with proliferating cells representing the least committed, and cells expressing cell cycle exit and p53 pathway genes the most committed to adipogenesis. This aligns with previous findings indicating that growth arrest is a prerequisite for adipogenic commitment in human cells.^43^

Adipose progenitors in cluster p2 express TNF-α-responsive markers such as *ICAM1* and *CXCL8*, resembling hASC clones that exhibit an enhanced thermogenic response upon cyclic AMP (cAMP) stimulation, and have been proposed to represent beige progenitors.^44^ However, our paired scRNA-seq datasets during early adipogenesis reveal striking similarity between white and beige differentiation trajectories, suggesting a dual white and beige potential for this progenitor population. Consistently, sorted ICAM^hi^ hASCs are equally competent for white and beige adipogenesis as the total, unsorted hASC population. These results support a model in which white and beige adipocytes represent distinct states along a shared differentiation trajectory rather than separate lineages. Indeed, Lazarescu et al. recently proposed that “classical” (white) adipocytes arise through the loss of specialized functions along a pseudo-temporal continuum,^22^ a concept that aligns with our recent findings that white adipocytes display a global decrease in three-dimensional chromatin interactions relative to beige adipocytes.^37^ Together, these human data indicate that white and beige adipocytes exist along a phenotypic continuum, with beige cells representing an inducible functional state that can emerge from, or revert to, a white phenotype in response to environmental cues. This model is further supported by mouse studies showing that white adipocytes can transdifferentiate into beige cells^45^ and subsequently revert to a white state.^46^^,^^47^

Our fine-mapping of early adipogenesis shows a rewiring of the ECM transcriptome 24h post-induction, recapitulating the transcriptomes of multipotent SWAT cells, initially described at day 3 of adipogenesis.^18^^,^^19^ Interestingly, our TSS-level analysis reveals an increased proportion of SWAT cells upon beige induction, and a distinct ECM genes expression profile of beige-treated SWAT cells. This supports the notion that ECM remodeling by SWAT cells in response to beiging stimuli might play a key role in promoting the thermogenic phenotype of mature adipocytes.^48^ Conversely, defective remodeling of the ECM in obese brown adipose tissue compromises thermogenic activation,^49^ highlighting the importance of this process in enabling adipose tissue functional plasticity.

By 3 days of adipogenic stimulation, cells adopt a spectrum of states characterized by a gradual upregulation of genes involved in fatty acid metabolism, mitochondrial matrix, and oxidative phosphorylation, ultimately leading to the robust induction of the adipogenic program. Along this trajectory, beige-induced cells show an upregulation of fatty acid synthesis enzymes, consistent with the early-onset adipogenic response described in brown depot-derived hASCs.^20^ These observations raise the possibility that these early fate-specific transcriptional programs may be driven by the differential expression of chromatin modifiers and transcriptional regulators observed at an earlier time point. Beige-induced cells also display an increased expression of protein folding chaperones and reduced expression of ribosomal protein genes, pointing to fate-specific translational regulation. In agreement, the modulation of translation efficiency drives the selective down-regulation of oxidative phosphorylation transcripts and enhanced translation of tricarboxylic acid (TCA) cycle mRNAs during murine beige adipogenesis.^50^ Collectively, these observations suggest that early modulations of transcriptional and translational regulators may influence the trajectory of progenitors toward a mature beige adipocyte phenotype.

Large interindividual differences in adaptative thermogenesis have been documented in humans.^51^^,^^52^ While hASCs of all six subjects examined here show a robust induction of a beige adipocyte transcriptional program upon PPARγ agonist treatment,^27^ the magnitude of UCP1 induction and activation of non-canonical thermogenic pathways markedly vary between individuals. Because these subjects display broadly similar hASC populations, our data argue against a direct link between a progenitor subtype abundance and thermogenic potential. Instead, interindividual variation in thermogenic mechanisms may arise from differences in regulatory priming, including variations in chromatin states at thermogenic gene loci. Such variation may arise from genetic variation in PPARγ-binding sites,^53^ or from an epigenetic memory of past adipose tissue stress.^54^ Consistent with this model, transient inhibition of SUMOylation in hASCs imprints a beiging-prone transcriptional state that persists throughout differentiation,^55^ underscoring the importance of epigenetic priming in shaping human beige adipocyte fate.

Overall, this study provides a high-resolution characterization of early human adipogenesis by profiling over 70,000 primary cells across multiple donors and differentiation conditions. We identify transcriptionally similar trajectories for early white and beige adipocyte differentiation that trace back to a shared pool of committed progenitors with dual differentiation potential. Consistent with this model, hASCs from human white and brown adipose depots also show overlapping trajectories when differentiated under similar conditions.^18^^,^^20^

### Limitations of the study

While transcriptomic trajectories capture commitment dynamics, they do not resolve regulatory states encoded at the chromatin or post-transcriptional level, which likely influence thermogenic output. In addition, integration across time points in the trajectory analysis may limit sensitivity to treatment-dependent divergence between white and beige programs. Future studies incorporating multiple donors profiled across the full differentiation time course could help resolve this question. Nevertheless, our analysis provides a comprehensive resource for exploring human adipose cell states and plasticity, for example, in the context of pathological adipose tissue remodeling.

## Resource availability

### Lead contact

Requests for further information and resources should be directed to and will be fulfilled by the lead contact, Nolwenn Briand (nolwenn.briand@medisin.uio.no).

### Materials availability

This study did not generate new unique reagents.

### Data and code availability

• Bulk RNA-seq datasets are available at Gene Expression Omnibus under accessions NCBI GEO: GSE256259 and GSE293133. scRNA-seq data have been deposited at GEO under accession NCBI GEO: GSE301263 and are publicly available as of the date of publication (see key resources table).
• All original code has been deposited at Zenodo and is publicly available at https://doi.org/10.5281/zenodo.23272450 as of date of publication.
• Any additional information required to reanalyze the data reported in this paper are available from the lead contact upon request.

## Acknowledgments

This work was funded by the 10.13039/501100005366University of Oslo, South-East Health Norway grant no. 40202 to P.C., 10.13039/501100005416Research Council of Norway grant no. 313508 to P.C., and Nansen Fund 17368 to N.B. We thank Anita Løvstad Sørensen for expert technical assistance and the Genomics Core Facility (10.13039/100031365Oslo University Hospital) for professional services. We thank the MolMed Imaging Platform (MIP) at the 10.13039/501100019110Institute of Basic Medical Sciences, University of Oslo for providing access to and training on relevant microscopes. We also thank Yan Zhang for assistance with FACS experiments and James Wilmouth for input on FACS analysis.

## Author contributions

S.H.P. and N.B. conceived and designed the study. S.H.P. performed bulk and scRNA-seq analyses. D.D. performed immunostainings, image analyses, and FACS experiments. N.M.G. prepared cells for scRNA-seq. N.B. performed immunoblotting. D.D., N.M.G., and P.C. revised the manuscript. S.H.P. and N.B. wrote the manuscript.

## Declaration of interests

The authors declare no competing interests.

## STAR★Methods

### Key resources table

**Table:** 

REAGENT or RESOURCE	SOURCE	IDENTIFIER
**Antibodies**		

FAS	Santa Cruz, sc-48357	RRID: AB_627584
PPARγ	Thermofisher, MA5-14889	RRID: AB_10985650
γTubulin	Sigma, T5326	RRID: AB_532292
ICAM1	BioLegends, HA58	RRID: AB_10916103
PE Mouse IgG1, κ	BioLegends, MOPC-21	RRID: AB_326435

**Deposited data**		

Bulk RNAseq (day15)	Hazell Pickering et al.^27^	NCBI GEO: GSE256259
Bulk RNAseq (day1 and 3)	This paper	NCBI GEO: GSE293133
Single cell RNAseq	This paper	NCBI GEO: GSE301263

**Oligonucleotides**		

*Fw_ICAM1*	CGTGAATGTGCTCTCCCCC	Invitrogen
*Rv_ICAM1*	TCCCTTTTTGGGCCTGTTGT	Invitrogen
*Fw_CXCL8*	GCAGCTCTGTGTGAAGGTGC	Invitrogen
*Rv_CXCL8*	TCTCAGCCCTCTTCAAAAACTTC	Invitrogen
*Fw_PPARG1*	TGACCAGAAGCCTGCATTTCT	Invitrogen
*Rv_PPARG1*	AAGGCTGACTCTCGTTTGAG	Invitrogen
*Fw_PPARG2*	AAGCGATTCCTTCACTGATACAC	Invitrogen
*Rv_PPARG2*	CTTCCATTACGGAGAGATCCAC	Invitrogen
*Fw_PLIN1*	GACAAGGAAGAGTCAGCCCC	Invitrogen
*Rv_PLIN1*	GAGAGGGTGTTGGTCAGAGC	Invitrogen
*Fw_UCP1*	CTCACCGCAGGGAAAGAA	Invitrogen
*Rv_UCP1*	GGTTGCCCAATGAATACTGC	Invitrogen
*Fw_CIDEA*	TGAAGGCCACCATGTATGAG	Invitrogen
*Rv_CIDEA*	CAGGAACCGCAGCAGACT	Invitrogen
*Fw_PM20D1*	GACCGTACAGTCCGTCTTCC	Invitrogen
*Rv_PM20D1*	GAATCGGCTGTCTGTGTTGC	Invitrogen
*Fw_SF3A1*	AGGGTCCAGTGTCCATCAAA	Invitrogen
*Rv_SF3A1*	AGAGACCTGGTCCGTGAGTG	Invitrogen

**Software and algorithms**		

fastp (read quality filtering)	Chen et al. https://github.com/OpenGene/fastp	
HISAT2 (read alignment to hg38/GENCODE 32)	Kim et al. https://daehwankimlab.github.io/hisat2/	2.2.1
featureCounts (read counting)	Liao et al. https://subread.sourceforge.net/	Subread v1.5.3
edgeR (filterByExpr & TMM normalization)	Robinson et al. https://bioconductor.org/packages/edgeR	
limma-voom (eBayes method for differential expression)	Law et al.^56^https://bioconductor.org/packages/limma	
RUVSeq (remove unwanted variation)	Risso et al. https://bioconductor.org/packages/RUVSeq	*v1.34.0*
Cell Ranger (scRNA-seq read counting)	10× Genomics https://www.10xgenomics.com/support/software/cell-ranger	*v7.0.0*
Seurat (single-cell analysis)	Hao et al.^57^https://satijalab.org/seurat/	*v5.0.3*
clustree (clustering resolution visualization)	Zappia and Oshlack https://github.com/lazappi/clustree	*v0.5.1*
*SCAFE (TSS quantification from 5′ scRNA-seq)*	Moody et al.^40^https://github.com/chung-lab/SCAFE	*v1.0.0*
*Monocle3 (trajectory analysis)*	Cao et al.^58^https://cole-trapnell-lab.github.io/monocle3/	*v1.3.7*
*SeuratWrappers*	Satija Lab https://github.com/satijalab/seurat-wrappers	*v0.3.2*
*sceasy (Seurat/AnnData format conversion)*	https://github.com/cellgeni/sceasy	*v0.0.7*
*schard (AnnData to Seurat conversion)*	https://github.com/koenvandenberge/schard	*v0.0.1*
*scVI (deep generative model for scRNA-seq)*	Lopez et al.^35^https://scvi-tools.org/	*v1.3.3*
*Genes2Genes (pseudotime trajectory comparison)*	Sumanaweera et al.^42^https://github.com/Teichlab/Genes2Genes	*v0.2.0*
*scanpy (Python single-cell analysis)*	Wolf et al. https://scanpy.readthedocs.io/	*v1.10.3*
*optbinning (optimal pseudotime binning)*	https://github.com/guillermo-navas-palencia/optbinning	*v0.21.0*
*velocyto (RNA velocity unspliced/spliced counting)*	La Manno et al.^59^http://velocyto.org/	*v0.17.17*
*velociraptor (RNA velocity estimation)*	Rue-Albrecht et al.^60^https://bioconductor.org/packages/velociraptor	*v1.8.0*
*clusterProfiler (GSEA and overrepresentation analysis)*	Wu et al. https://bioconductor.org/packages/clusterProfiler	*v4.6.2*
*MSigDB (gene set collections)*	Liberzon et al.https://www.gsea-msigdb.org/gsea/msigdb/	*v2024*
*FlowCore (FACS data analysis)*	Hahne et al. https://bioconductor.org/packages/flowCore	*v2.10.0*
*tinyatlas gene sets (cell cycle scoring)*	https://github.com/hbc/tinyatlas	
*QuPath (immunofluorescence image analysis)*	Bankhead et al. https://qupath.github.io/	*v0.4.3*
*ImageJ*	Schneider et al. https://imagej.nih.gov/ij/	
*Image Lab (densitometry)*	Bio-Rad https://www.bio-rad.com/en-us/product/image-lab-software	

**Other**		

Illumina NovaSeq (bulk RNA-seq and scRNA-seq sequencing)	Illumina	N/A
10× Genomics Chromium (GEM-X; scRNA-seq library preparation)	10× Genomics	N/A
BD FACS Aria IIu (cell sorting for scRNA-seq)	BD Biosciences	N/A
BD FACSMelody cell sorter (ICAM1 sorting and flow cytometry)	BD Biosciences	N/A
Andor Dragonfly spinning disk confocal (immunofluorescence imaging)	Andor	N/A

### Experimental model and study participant details

Primary ASCs were isolated from subcutaneous fat obtained by liposuction from six unrelated female donors (28 ≤ age (years) ≤ 50; 20.90 ≤BMI (kg/m^2^) ≤ 29.02; Table S1) after informed consent (approval by the Regional Committee for Research Ethics for Southern Norway, REK 2013/2102 and 2018–660). As all six donors were female, the influence of sex on the results was not assessed. Liposuction material was washed repeatedly in HBSS (Gibco) supplemented with 1% penicillin-streptomycin (Gibco) and 2.5 μg/mL fungizone (Gibco) then digested in DMEM/F12 containing 1% type II collagenase (Worthington) at 37°C for 50 min. Following centrifugation, the stromal vascular fraction was separated from mature adipocytes, washed, filtered through 100 μm strainers, and plated in DMEM/F12 (17.5 mM glucose) supplemented with 10% FBS, 1% penicillin/streptomycin, and 20 ng/mL FGF2 (proliferation medium). After 24 h, non-adherent cells were removed, and adherent hASCs were expanded under standard culture conditions. All primary cells used in this study were tested negative for mycoplasma contamination. For differentiation, hASCs were seeded at high density in proliferation medium. Upon confluency, fibroblast growth factor was removed, and cells cultured for 72 h in DMEM/F12 (17.5 mM glucose) with 10% fetal calf serum (basal medium) before induction of differentiation (day 0). For white adipose differentiation, hASCs were induced with a cocktail of 0.5 μM 1-methyl-3 isobutyl xanthine (Sigma, I5879), 1 μM dexamethasone (Sigma, D4902), 10 μg/mL insulin (Sigma, I9278) and 200 μM indomethacin (Sigma, I7378-56) in basal medium. Differentiation media was renewed every 3 days until day 9, after which cells were maintained in DMEM/F12 (17.5 mM glucose) with 10% fetal calf serum and 10 μg/mL insulin. For beige adipose differentiation, media were supplemented with 1 μM Rosiglitazone (Sigma, R2408) from day 0 until day 15. Samples were harvested on D0, then D1, D3, and D15 after induction. All differentiation experiments were done in at least three biological replicates with hASCs at passage 3–8. Cells from each donor were divided and differentiated in parallel under white and beige conditions, so that each donor served as its own control. No randomization or blinding was applied.

### Method details

#### Bulk RNA sequencing and analysis

Bulk RNA-seq was conducted as previously described.^27^ In summary Total RNA was isolated from samples harvested at differentiation endpoint (day 15) using the RNeasy kit (QIAGEN) for 6 independent subjects. PolyA-selected RNA was sequenced from paired-end libraries (TruSeq Stranded mRNA kit; Illumina) using Novaseq platform (Illumina). Reads were filtered with fastp, aligned to the hg38 genome (GENCODE 32 annotation) with hisat2, and counted using featureCounts. Low abundance genes were filtered using filterByExpr from edgeR and then normalized using the trimmed mean of M values method. At day 1 and 3 remove unwanted variation (RUV; v1.34.0) was used to normalize between experiments and then beige and white gene expression was compared pairwise for each time point using the robust eBayes method with limma-voom adjustment (hereafter eBayes test.^56^; At day 15, a combined test which concatenated the white versus beige eBayes tests per subject was also used to assess if each gene was significantly different between white and beige conditions in any of the subjects. Subjects were compared to the mean of the remaining subjects using the eBayes test (subject vs. all subjects). *p*-values were adjusted by false discovery rate.

#### Single cell RNA-seq

##### Cell isolation and library preparation

Cells were harvested using Trypsin on days 0, 1 and 3 of differentiation, washed in PBS containing 2% BSA and sorted in a BD FACS Aria IIu flow cytometer to ensure a single-cell suspension without debris or apoptotic cells. Final concentration of cells after sorting was adjusted to 850–1000 cells/μL buffer, to load a total of 30000 cells with ≥90% viability in the 10× Genomics Chromium platform, aiming to sequence 10 000 cells per sample. For single-cell 5′ RNA sequencing, barcoding and library preparation was performed using GEM-X technology, and sequencing was performed by Illumina NovaSeq at approximately 50000 reads per cell.

##### Data analysis

Reads from each sample were counted (excluding introns) using Cellranger (v7.0.0) then aggregated for each analysis set. For progenitors and TSS-level analysis of adipogenesis both read pairs were considered, while in the gene-level analysis of adipogenesis 5′ read2 only mode was used.

Cells were required to have maximum fraction of mitochondrial reads < median + three times the mean absolute deviation (progenitors = 2.44%, adipogenesis = 5.36%) and minimum 2000 detected transcripts. Cells were randomly downsampled to equalize cell number between time points in white adipogenesis and between beige and white conditions in paired adipogenesis. Top 2000 variable features were selected for principal component analysis, carrying 20 dimensions forward for Louvain graph-based clustering (resolution 0.5), UMAP (n.neighbors = 20 for adipogenesis) and TSNE dimensionality reduction in Seurat v5.0.3.^57^ For progenitors, Louvain clustering at resolutions between 0.1 and 0.8 were visualized using clustree (v0.5.1) and based on cluster stability, marker genes and gene set analysis was compared for resolutions 0.3 and 0.5. Wilcoxon test was used to find all positive marker genes per cluster (min logfc 0.5) or subject (min logfc 0.25) with prefiltering for feature detection (min.pct 0.25). Pairwise beige vs. white expression tests used default parameters (min logFC 0.1 & min.pct 0.01) with post hoc filtering for | log2FC | > 0.25 & pct.1 + pct.2 > 0.2. For all tests Bonferroni adjusted *p* value <0.01. RPCA, FastMNN and Harmony integration were implemented by the IntegrateLayers function from Seurat. The SCVI model using the negative binomial distribution was established with 30 latent dimensions and 3 hidden layers and then trained with a batch_size of 256 (scvi v1.3.3). Sceasy v0.0.7 was used to convert to python anndata format and schard (v0.0.1) to convert back to Seurat format.

SCAFE was used to cluster, filter and quantify the usage of TSSs derived from alignments of 5′ scRNA-seq reads (v1.0.0).^40^ SCAFE workflow was run in solo mode for each sample and then samples were aggregated to get a common set of CREs prior to TSS filtering (default model, robust threshold). On day 1, only replicate 1 was used and cells with ≥1000 detected TSSs were analyzed with Seurat as detailed above.

Monocle3 (v1.3.7;^58^) analysis was run separately for white and beige trajectories: Seurat objects were split to include only day 1 and day 3 cells from the relevant condition, in addition to day 0 cells. Variable genes were independently determined, seurat objects were converted to monocle3 format with SeuratWrappers (v0.3.2), and a trajectory graph was learned on the fastMNN integrated UMAP (close loop = T). Cells were ordered for pseudotime by setting the closest vertex to the center of the adipogenic cluster (b4/t4) as the root node. To generate pseudotime boxplots, trajectory graphs and pseudotime were computed on 12 random subsets of 50% or 3,300 cells per time point, whichever value was the smallest. After conversion to anndata format, Genes2Genes (v0.2.0;^42^) was used to compare pseudotime scores between trajectories based on the union of the top 2000 variable genes for each trajectory (as calculated by scanpy; v1.10.3). The python package optbinning (v0.21.0) was used to determine the optimal number of pseudotime bins.

Unspliced and spliced reads were counted for RNA velocity analysis with velocyto (v 0.17.17;^59^) with paired end reads where possible. Highly variable genes with detectable unspliced and spliced reads and more than 20 shared reads were used to estimate global RNA velocities with velociraptor (v1.8.0;^60^) using the dynamical model.^61^

Cell cycle was scored with CellCycleScoring function from Seurat with tinyatlas gene sets (https://github.com/hbc/tinyatlas).

#### Immunoblotting

Proteins were resolved on 12% SDS–PAGE, transferred onto nitrocellulose membranes (BioRad), and blocked with 5% BSA. Membranes were incubated for 1h at room temperature using the following antibodies: PPARγ (Thermofisher, MA5-14889), FAS (Santa Cruz, sc-48357) and γTubulin (Sigma, T5326). Proteins were visualized using IRDye-800 or HRP-coupled secondary antibodies. Bands were quantified by densitometry (Image Lab; BioRad) using γTubulin for normalization. Uncropped membranes are presented as Figure S13.

#### Immunofluorescence

Cells were grown on coverslips, fixed in 4% PFA for 10 min then permeabilised with Triton 0.1× for 15 min. After blocking for 1 h with 5% BSA in PBS, cells were incubated with primary antibodies overnight at 4°C and Alexafluor coupled secondary antibody for 1 h at room temperature. Images were acquired with Andor Dragonfly and analyzed with QuPath 0.4.3 software or ImageJ.

#### Fluorescence-activated cell sorting

Cells were grown in 10 cm dishes until day 0 and trypsinised to a single cell suspension. The suspension was filtered through a 40 μm mesh, diluted to 1 × 10 million cells/mL in PBS with 2% FBS and stained with phycoerythrin-conjugated rabbit anti-ICAM1 antibody (BioLegends, HA58) or isotype control (BioLegends, MOPC-21) both at a concentration of 1/100. Cells were incubated for 30 min at 4°C in the dark. After washing twice, cells were incubated with 1 μM DAPI (Sigma) to assess cell viability and processed through a BD FACSMelody cell sorter. Debris, doublets and dead cells (highlighted by DAPI staining) were discarded. Isotype-stained cells were used to assess background fluorescence and define ICAM1-negative (ICAM1^-^) population and the 10% of cells with the highest ICAM1 staining intensity were classified as ICAM1^hi^. Finally, live cells were processed through the FACS to obtain the unsorted population. Measurements were acquired on the BD FACSMelody cell sorter and analyzed using FlowCore package (v2.10.0). ICAM1^hi^, ICAM1^-^, and unsorted hASCs were plated at confluency and cultured for 72 h in DMEM/F12 (17.5 mM glucose) with 10% fetal calf serum (basal medium) before induction of white or beige differentiation.

#### RT-qPCR

RNA was isolated using RNeasy kit (QIAGEN) and 500 ng was used for cDNA synthesis using High-Capacity cDNA Reverse Transcription Kit (ThermoFisher). RT-PCR was done using IQ SYBR green (Bio-Rad Laboratories) with *SF3A1* as a reference gene. PCR conditions were 95°C for 3 min and 40 cycles of 95°C for 10 s, 60°C for 15 s, and 72°C for 20 s. PCR primers are listed in Table S10.

### Quantification and statistical analysis

Statistical analyses were performed in R and Python. Software, package versions and the functions used are listed in the key resources table and described in method details. The statistical test, the exact n, what n represents (subjects, independent experiments, cells or lipid droplets), the definition of center and the measures of dispersion and precision, and the meaning of every significance symbol are given in the figure legends. Each test is reported together with its multiple-testing correction. Differential expression in bulk RNA-seq was tested with limma-voom eBayes (FDR-adjusted; white vs. beige: ∗∗∗ adjusted *p* < 0.0001, ∗∗ adjusted *p* < 0.001, ∗ adjusted *p* < 0.01; subject vs. all subjects: §§ adjusted *p* < 0.0001, § adjusted *p* < 0.001). Marker genes and beige vs. white differences in scRNA-seq were tested with the Wilcoxon test, treating cells as replicates (Bonferroni-adjusted *p* < 0.01). Proportions of white and beige cells per TSS cluster were compared with Chi-squared tests (∗ FDR-adjusted *p* < 0.05, ∗∗ FDR-adjusted *p* < 0.01). GSEA (minGSSize = 3, eps = 1 × 10^-40^) and Overrepresentation tests were run with ClusterProfiler (v4.6.2;^62^) and FDR adjusted *p*-values were used for sorting. To keep *p*-values comparable between analyses, statistical tests included all gene sets from the Hallmark, Gene Ontology, WikiPathways, KEGG and Reactome libraries at MSigDB (v2024;^63^). To reduce redundancy, only selected ontology libraries are shown per figure. RT-qPCR and lipid droplet area were compared with the Kruskal-Wallis test. Western blot densitometry was analyzed by two-way ANOVA with Tukey’s multiple comparisons test (∗∗∗*p* < 0.001, ∗∗*p* < 0.01).

## Supplemental information

Document S1. Figures S1–S13

Table S1. Human subjects characteristics, related to Figure 1

Table S2. Enriched gene sets between subjects, related to Figure 2F

Table S3. Marker genes in RPCA integrated progenitor clusters, related to Figure 3B

Table S4. GO terms enriched in each progenitor cluster, related to Figure 3C

Table S5. Marker genes for clusters in white adipogenesis (a0–a11), related to Figures 4 and S4

Table S6. Cluster-specific differentially expressed genes between white and beige cells, related to Figure 5C

Table S7. Top differentially expressed annotated TSSs between white and beige cells in each TSS cluster, related to Figure 5H

Table S8. TSS-level alignment metrics from Genes2Genes comparison of white and beige adipogenic trajectories, related to Figure 6

Table S9. Gene-level alignment metrics from Genes2Genes comparison of white and beige adipogenic trajectories, related to Figure 6

Table S10. RT-qPCR primers, related to Figure 7
