# Supplementary material for: Fine mapping of early adipogenesis identifies committed progenitors with dual white and beige potential
Source: iScience. 2026 Oct 29;29(11):118086. doi: 10.1016/j.isci.2026.118086 (PMC13738169; doi:10.1016/j.isci.2026.118086)
Supplement: Supplementary file 1 [file mmc1.pdf]

iScience, Volume *29*

Supplemental information

Fine mapping of early adipogenesis identifies committed progenitors with dual white and beige potential

Sarah Hazell Pickering, Damien Dufour, Natalia M. Galigniana, Philippe Collas, and Nolwenn Briand

Supplementary figures

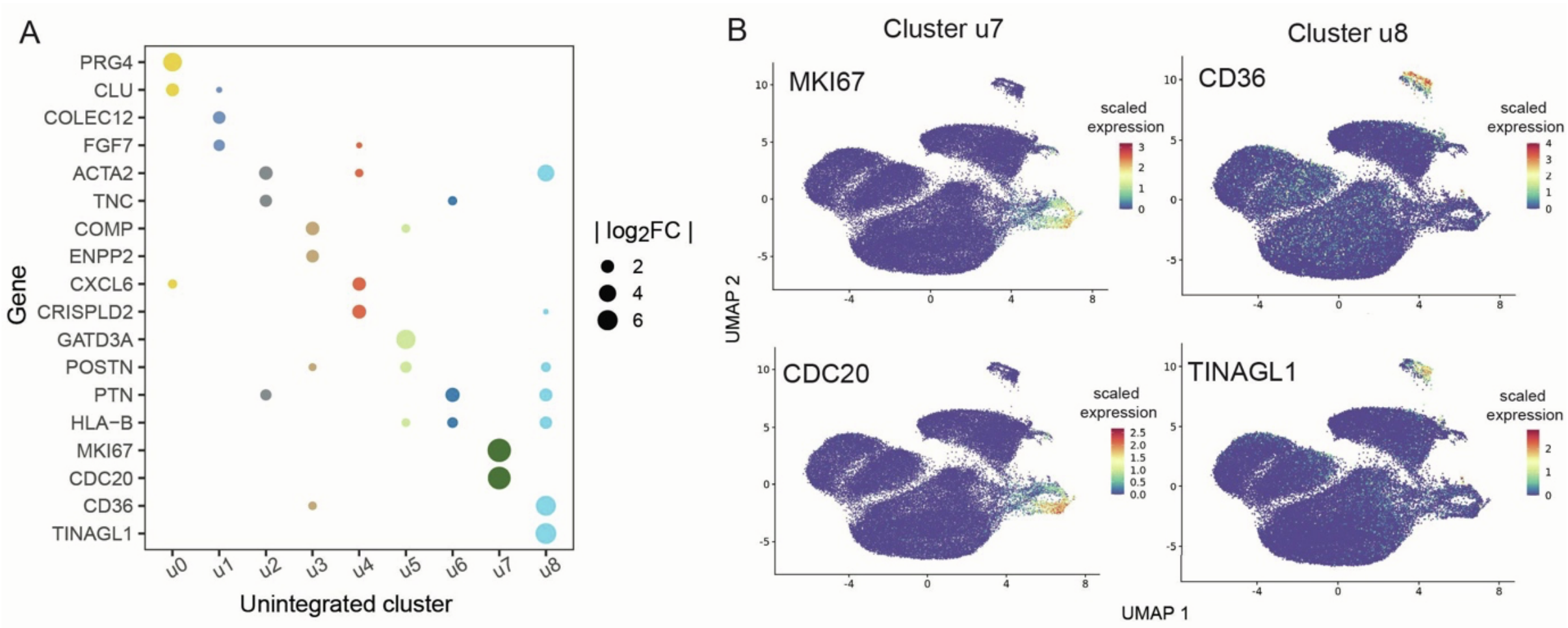

Figure S1: Markers of progenitors from unintegrated scRNA-seq in six subjects, related to Figure 1.
A Top 2 marker genes for each unintegrated progenitor cluster (u0-u8; adjusted $p < 0.01$, Wilcoxon test, cells as replicates). **B** Expression of top markers genes for clusters u7 and u8 visualized on integrated UMAP of 56,638 progenitor cells.

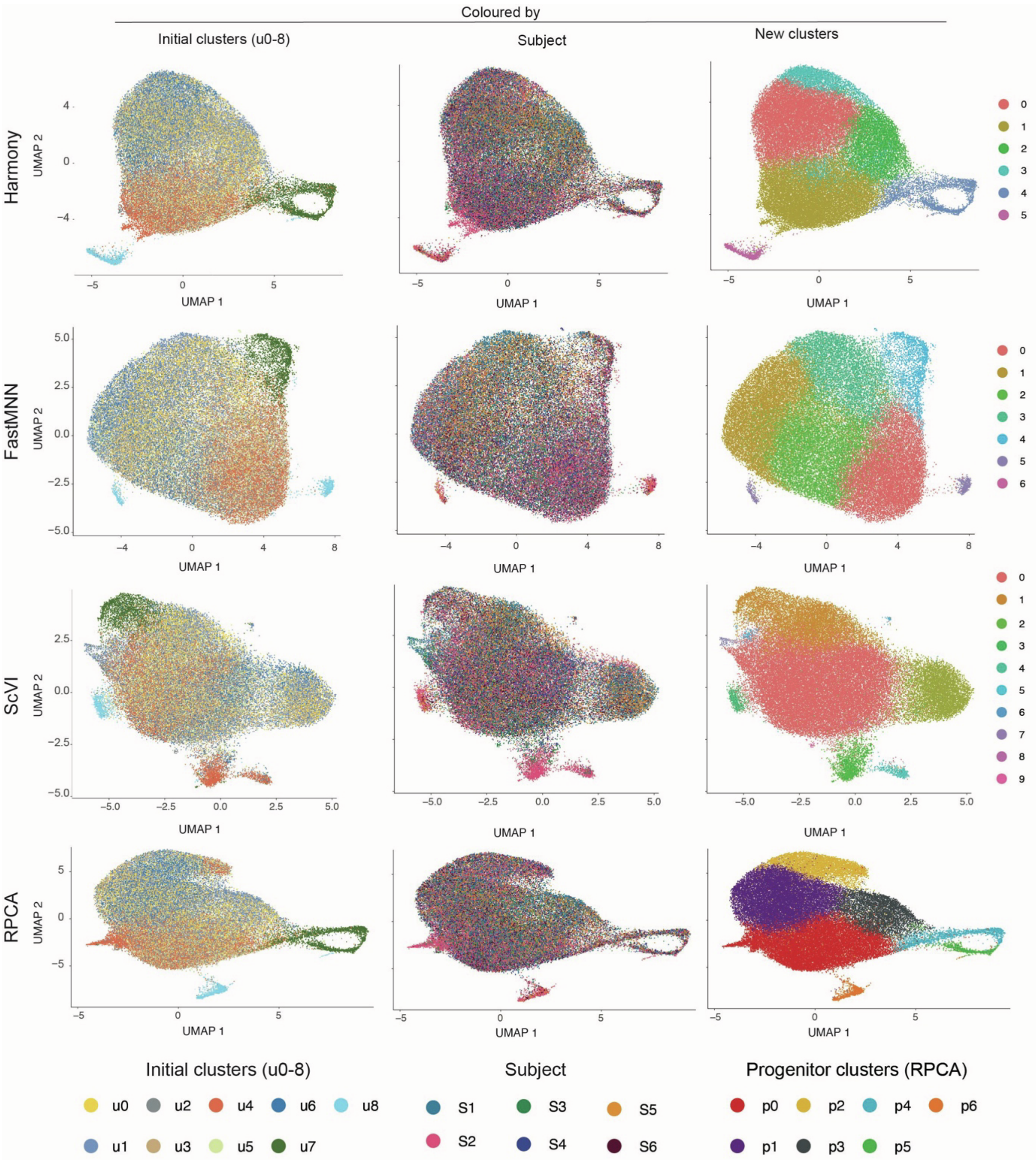

Figure S2: Integration methods in progenitor scRNAseq, related to Figure 3.
UMAPs of 56,638 progenitor cells after integration of progenitors by 4 methods (Harmony, FastMNN, ScVI RPCA; rows) and coloured by initial unintegrated clusters, subject and new integrated clusters (columns).

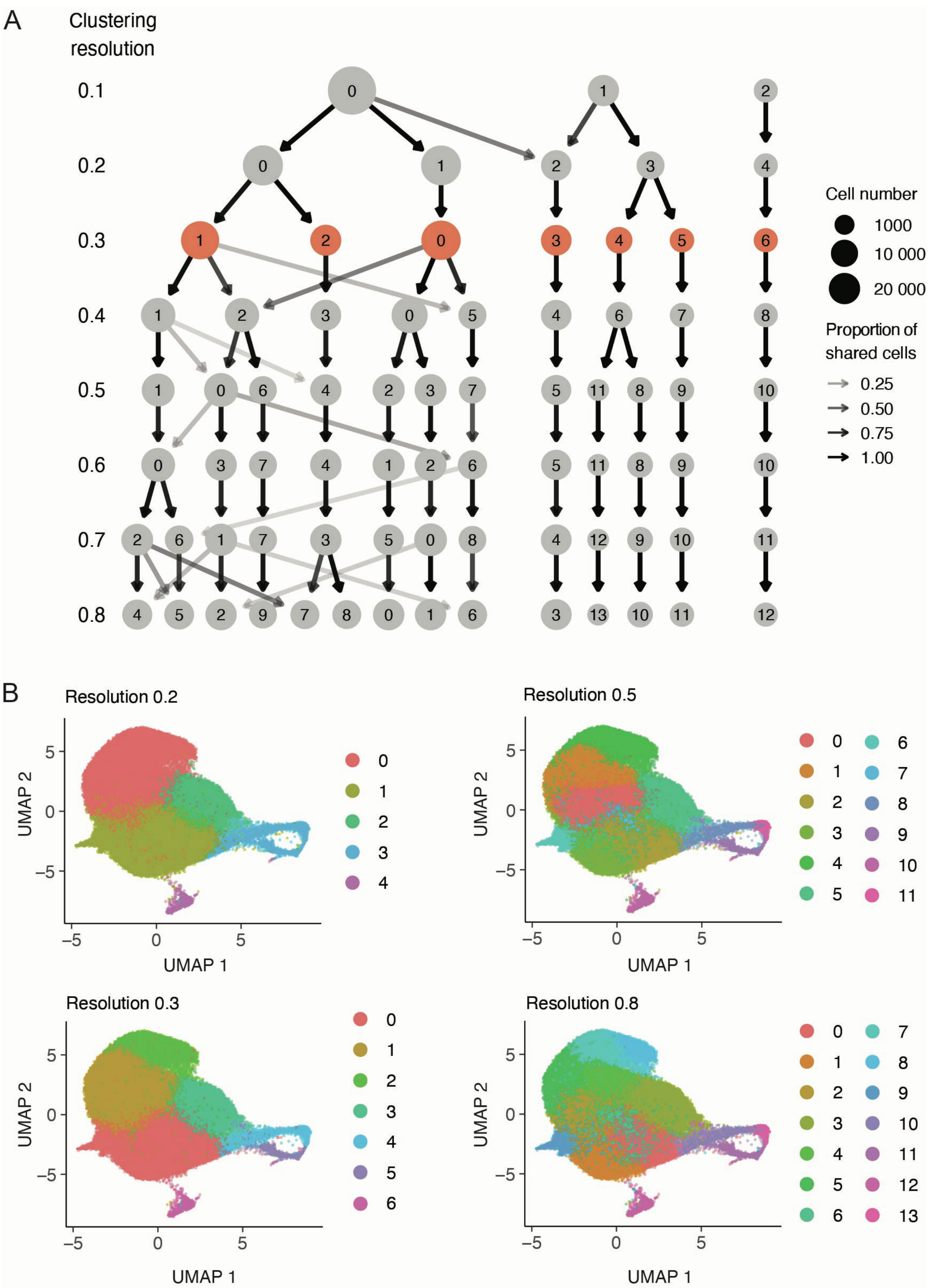

Figure S3: Clustering resolution trials in progenitor scRNAseq, related to Figure 3.
A Tree of cluster splits for clustering resolutions 0.1 - 0.8. Clustering at resolution 0.3 (orange) was selected for further analysis. **B** Louvain graph-based clusters shown on integrated UMAP of 56,638 progenitor cells at the indicated resolutions.

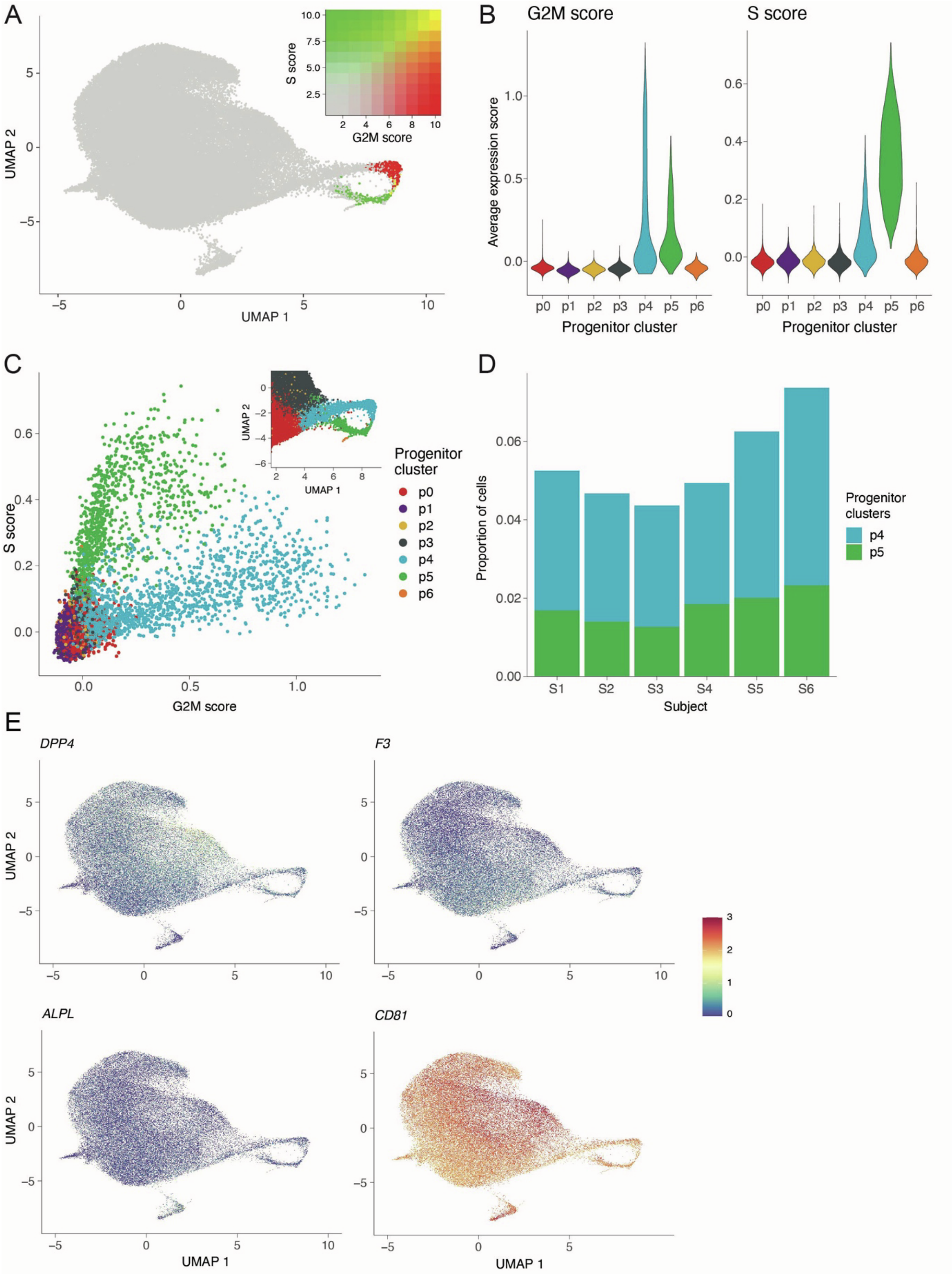

Figure S4: Cell cycle dynamics and cluster characterization in integrated adipose progenitors, related to Figure 3.
A G2M and S phase scores calculated as described by Tirosh et al. (2016) colored on RPCA integrated UMAP of 56,638 progenitor cells. **B** Violin plots of G2M and S phase scores in progenitor clusters. **C** Scatter plot of G2M and S phase colored by cluster. Inset shows cell cycle clusters (p5 & p6) on UMAP. **D** Proportion of subject cells in clusters. **E** Expression of hASC markers on RPCA-integrated progenitor UMAPs.

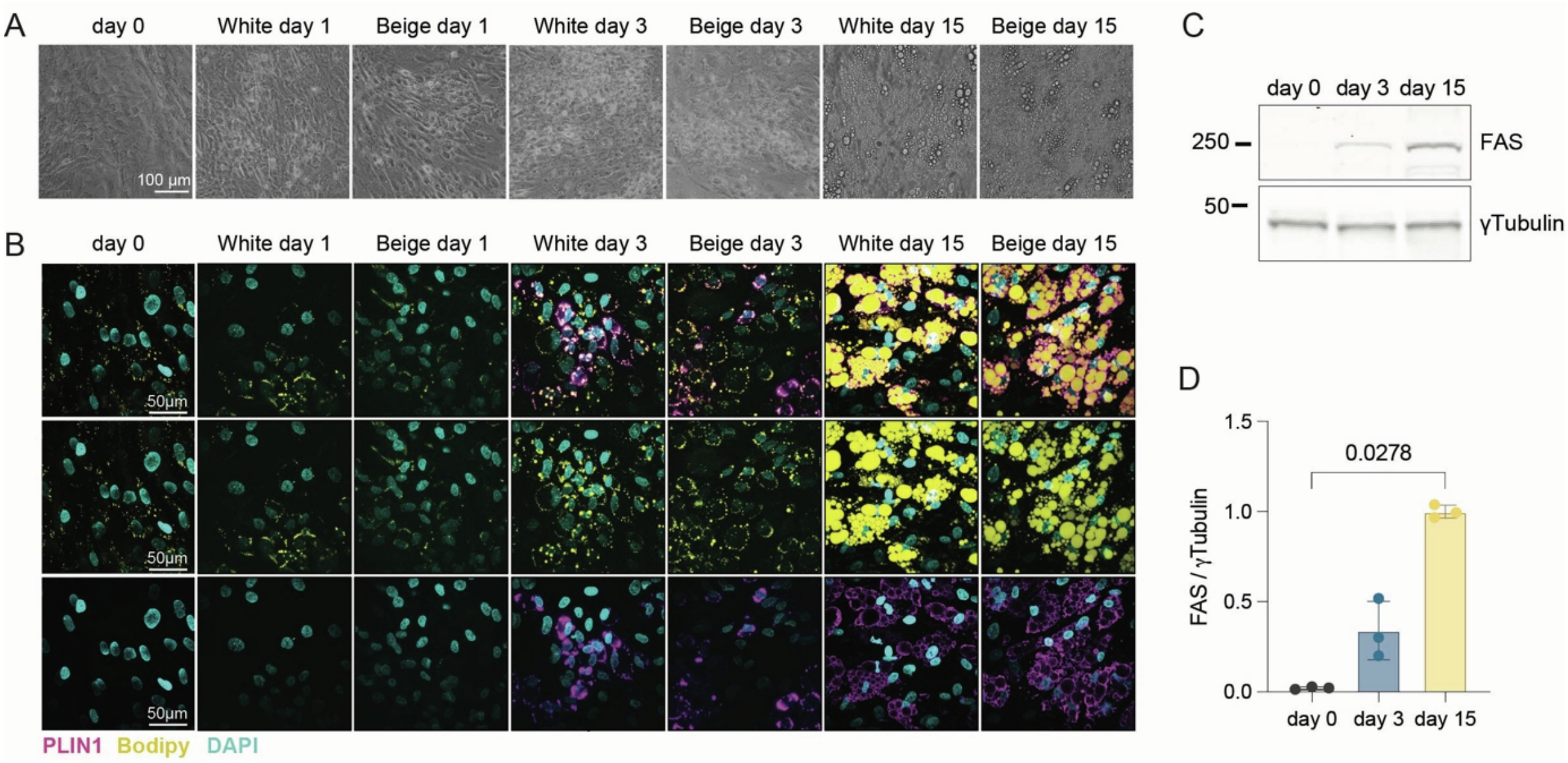

Figure S5: White and beige differentiation time course in subject S6, related to Figure 4.
A Phase contrast images during white and beige adipogenesis (representative images of n = 3 independent differentiation experiments; scale bar: 100 µm). **B** Immunofluorescence of Perilipin1 (PLIN1), Bodipy and DAPI stainings during white and beige adipogenesis (representative images of n = 3 fields per condition and per replicate, across 3 independent differentiation experiments; scale bar: 50 µm) **C** Western blot analysis of FAS expression at day 0, day 3 and day 15 of white adipogenesis. γTubulin is shown as a loading control. **D** FAS signals normalized to γTubulin, quantified from western blots (Mean with SD; Friedman test, n = 3 independent differentiation experiments).

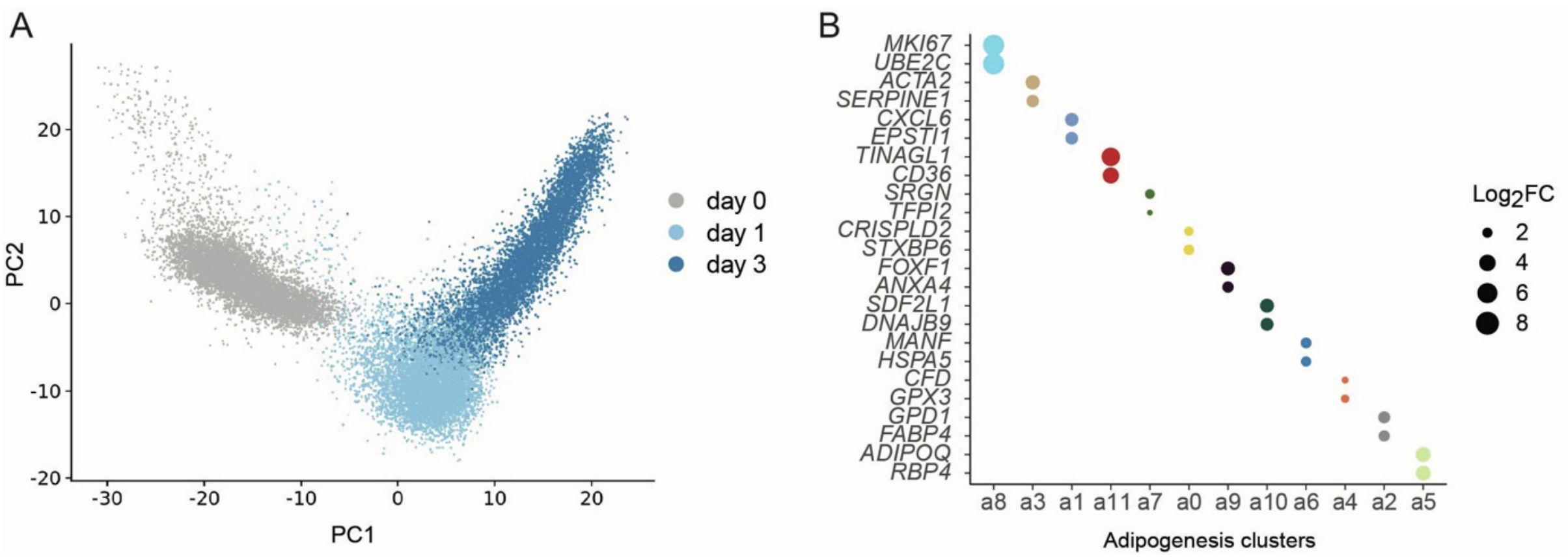

Figure S6: scRNA-seq clusters in early adipogenesis, related to Figure 4.
A PCA of 21,794 cells sampled from uninduced (day 0) and induced hASCs (day 1 and day 3) for scRNA-seq. **B** Top 2 marker genes for each Adipogenesis cluster (a0-a11).

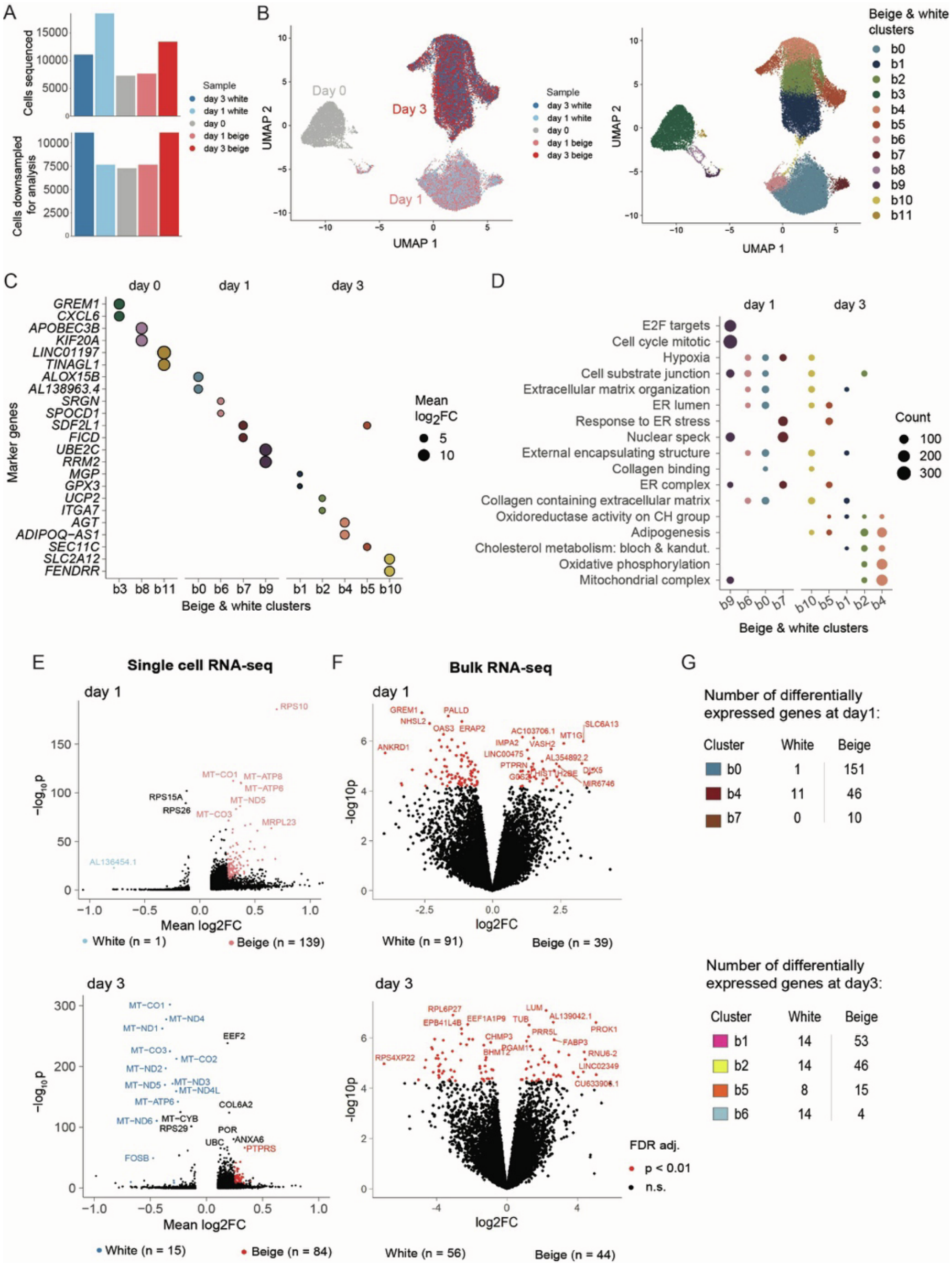

Figure S7: Early beige and white adipogenesis at the gene-level, related to Figure 5.
A Number of cells before and after downsampling for beige vs white comparisons. **B** UMAP of unintegrated scRNAseq in progenitors induced for white or beige differentiation, coloured by sample (left panel) and beige vs white clusters (right panel; b0-b12). **C** Dotplot of top 2 marker genes for each cluster ranked by log2FC (adjusted $p < 0.01$ & Log2FC > 0.5, Wilcoxon test, cells as replicates). **D** Top 2 enriched gene sets in marker genes of each cluster (FDR adjusted $p < 0.01$; Fischer's exact test). **E** Volcano plot for global beige vs white gene expression tests at day 1 (upper panel) and day 3 (lower panel) (adjusted $p < 0.01$ & | Log2FC | > 0.25 & min fraction of cells with expression 0.2; Wilcoxon test). **F** White vs beige differentially expressed genes from bulk RNA-seq at day 1 (left panel) and day 3 (right panel; FDR-adjusted $p < 0.01$; limma eBayes with RUV normalisation). **G** Tables of differentially expressed genes between beige and white cells within each cluster (adjusted $p < 0.01$ & Log2FC > 0.25 & min fraction of cells with expression 0, Wilcoxon test, cells as replicates).

.

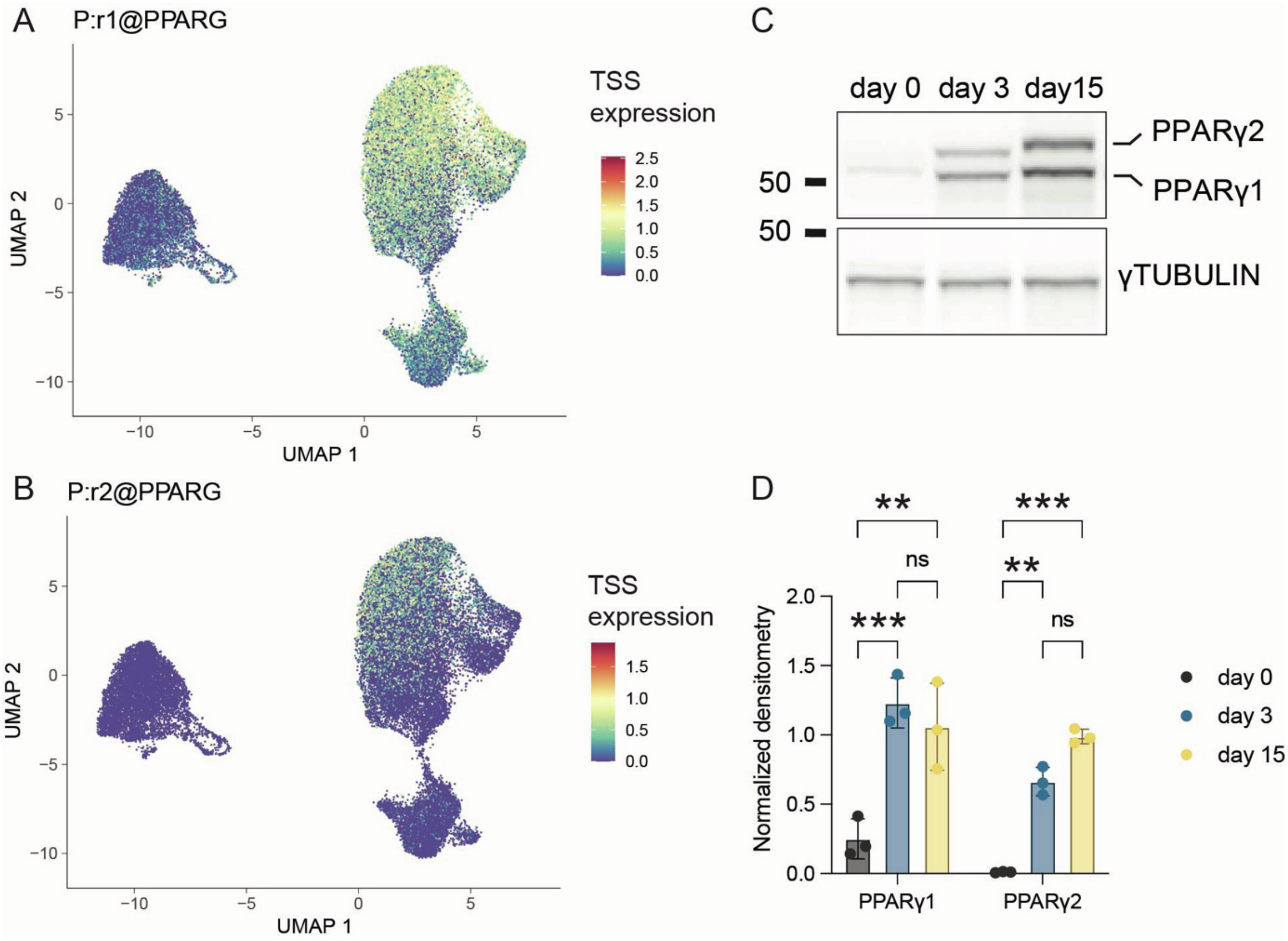

Figure S8: PPARG isoform switch in early adipogenesis, related to Figure 5.

A Scaled expression of PPARG1 (left panel) and PPARG2 (right panel) TSSs on UMAP of 33923 cells. **B** Western blot and **C** quantification of PPARγ1 and PPARγ2 expression normalized to γTubulin at day 0, day 3 and day 15 of white adipogenesis (***$p < 0.001$, **$p < 0.01$; Two-way ANOVA with Tukey's multiple comparisons test; n = 3 independent differentiation experiments).

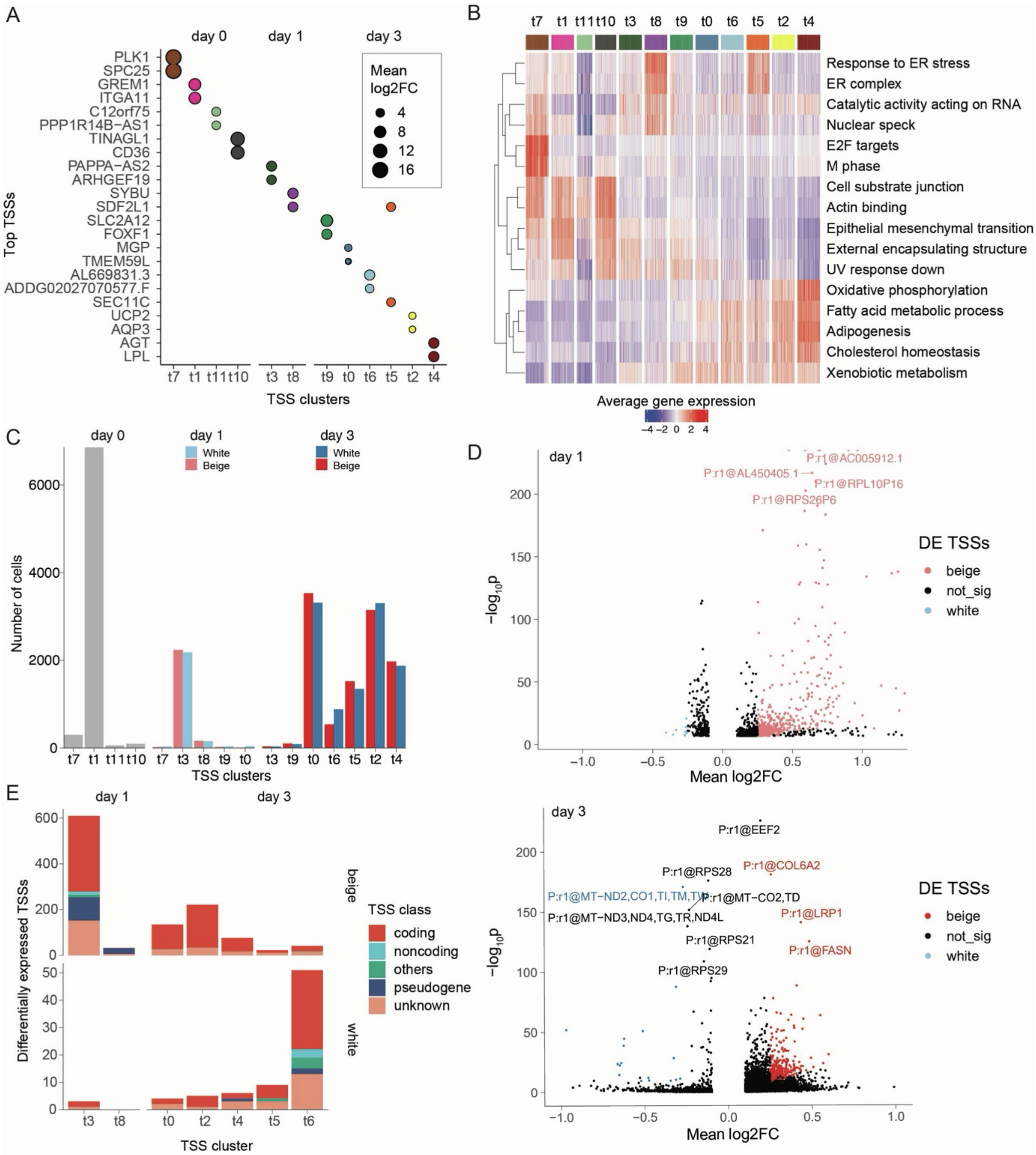

Figure S9: Early beige and white adipogenesis at the TSS-level, related to Figure 5.

A Top 2 marker genes per TSS-level cluster (t0-t11) ranked by log2FC (adjusted $p < 0.01$ & Log2FC > 0.5, Wilcoxon test, cells as replicates). **B** Heatmap of top 2 GO terms for each cluster ($p < 0.01$; Fischer's exact). **C** Number of cells from each condition in each TSS cluster. **D** Volcano plots for TSSs globally differentially expressed between white and beige cells at day 1 (top panel) and day 3 (bottom panel; Wilcoxon test with FDR adjusted $p < 0.01$ & |log2 FC| > 0.25). **E** Number of differentially expressed TSSs between white and beige cells within each cluster, separated by gene class.

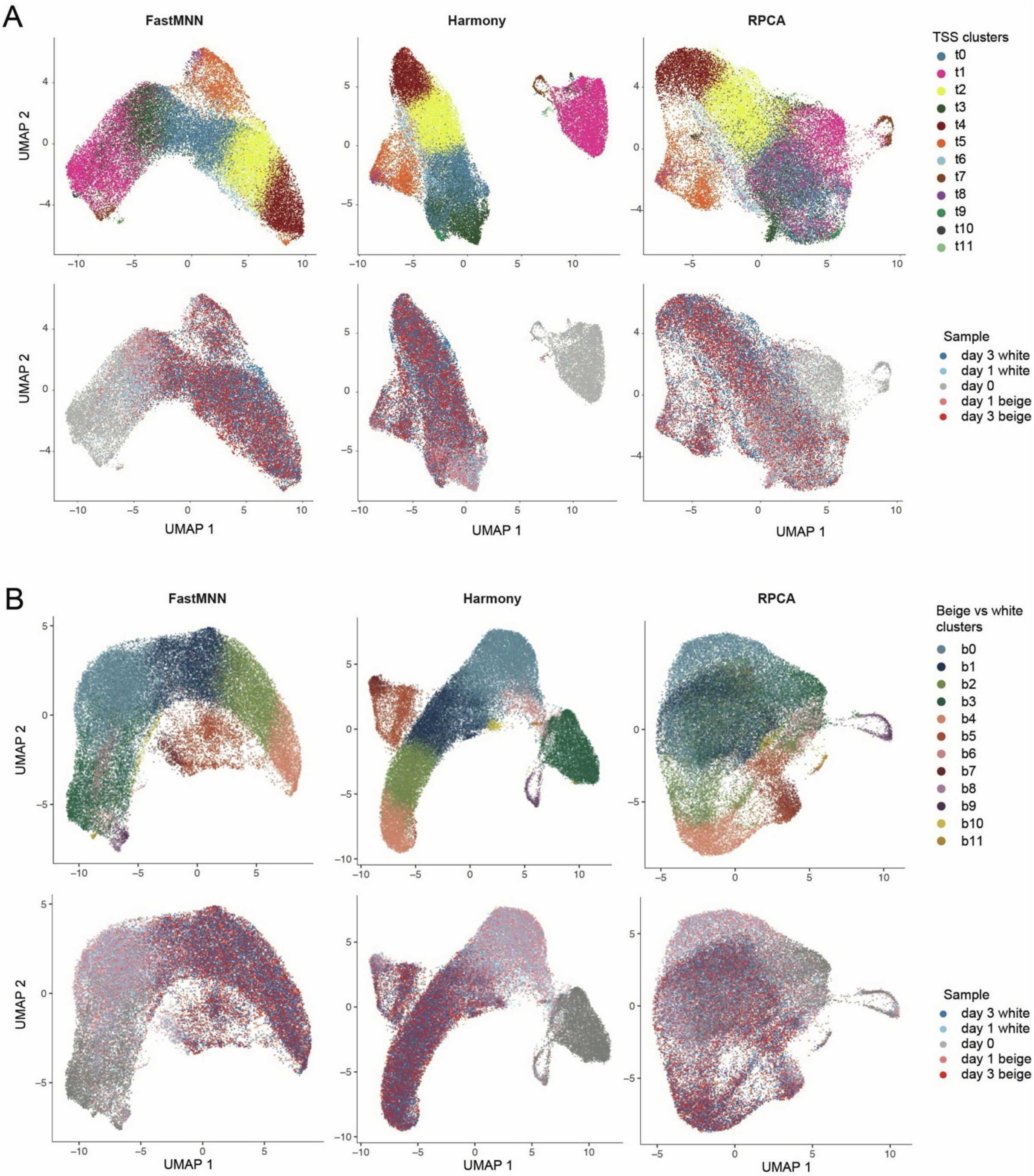

Figure S10: Integration strategies for white and beige differentiation trajectories, related to Figure 6.

A UMAP representations of TSS-level data integrated using FastMNN, Harmony or RPCA (columns). Cells (n=33 923) are colored by TSS-level clusters (t0-t11; top) or by samples (bottom). **B** UMAP representations of gene-level data integrated using FastMNN, Harmony or RPCA (columns). Cells (n=44 693) colored by beige vs white clusters (b0-b11; top) or by samples (bottom).

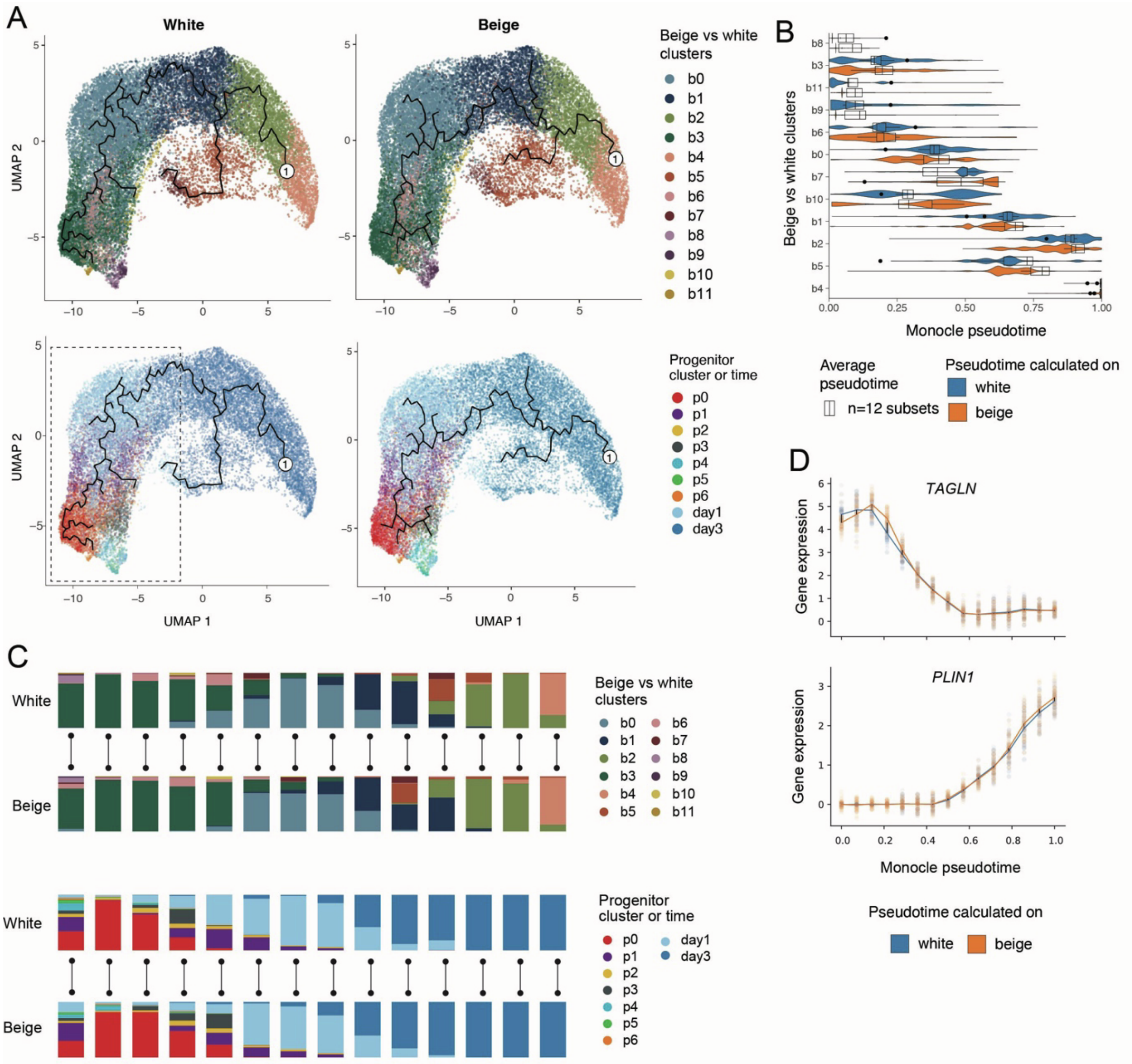

Figure S11: Progenitor trajectories on gene gene-level analysis of white and beige adipogenesis, related to Figure 6.
A Monocle3 trajectory graph of white or beige adipogenesis (columns) integrated at the gene-level with FastMNN. Cells (n=44 693) are coloured by beige vs white cluster (top) or progenitor cluster or time (bottom). Dashed-lined box corresponds to the inset in Figure 6D. **B** Pseudotime calculated for white and beige differentiation trajectories (Monocle3 on FastMNN UMAPs). Violin plots show cells within each Beige vs white cluster, boxplots the average pseudotime for each of 12 trajectory simulations on cell subsets. **C** Genes2Genes alignment of white and beige differentiation time-courses across 14 equally spaced pseudotime bins coloured by beige vs white clusters (top) or progenitor cluster or time (bottom); black lines represent matches between pseudotime bins. **D** Alignment of beige and white pseudotime trajectories for *TAGLN*, *ACTA2*, *PLIN1* and *FASN* genes (Genes2Genes).

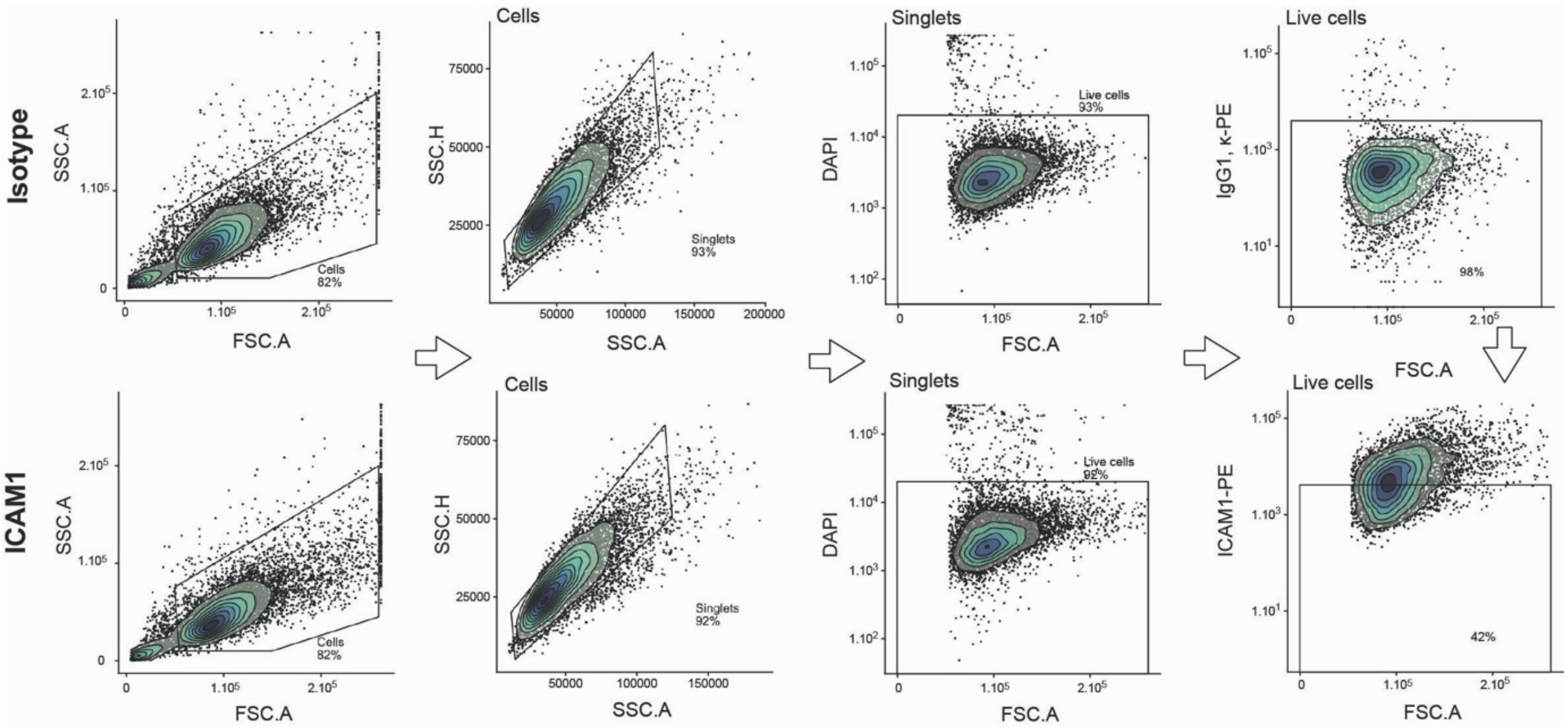

Figure S12: Gating strategy for the sorting of ICAM1^{hi} and ICAM1^{-} hASC subpopulations, related to Figure 7.
Representative gating from n = 4 independent sorting experiments

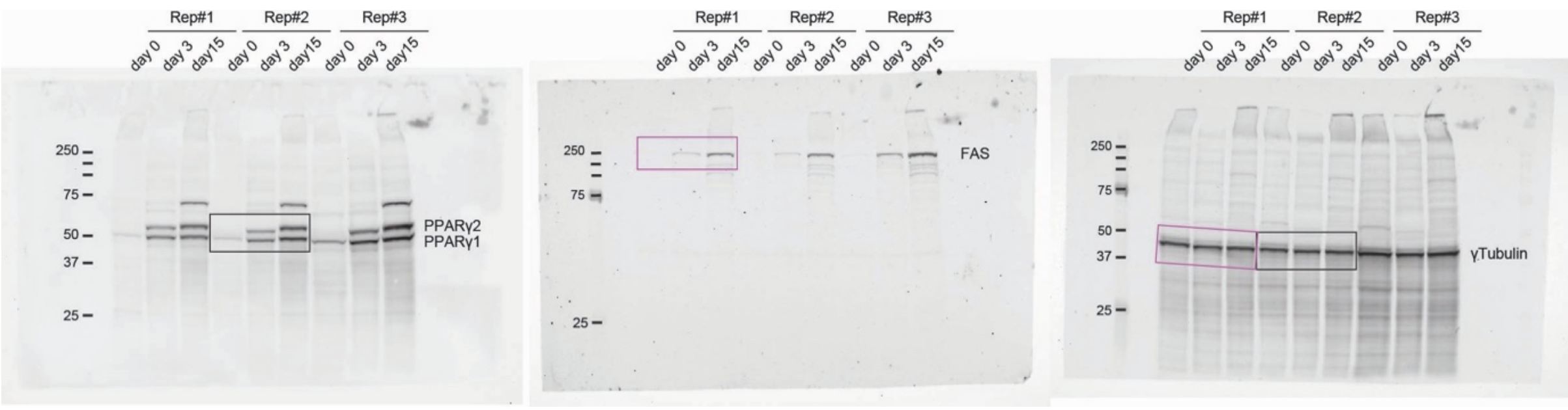

Figure S13: Uncropped western blot membranes, related to Figure 4,5.
Uncropped western blot replicates for **Supplementary Figure S5C,D & S8C,D**.
